# Possible Mechanisms of Resistance Development to Photodynamic Therapy (PDT) In Vulvar Cancer Cells

**DOI:** 10.3390/ijms232314689

**Published:** 2022-11-24

**Authors:** Beata Joanna Mossakowska, Anna Fabisiewicz, Barbara Tudek, Janusz Aleksander Siedlecki

**Affiliations:** 1 Department of Molecular and Translational Oncology, Maria Skłodowska-Curie National Research Institute of Oncology, 02-781 Warsaw, Poland; 2 Faculty of Biology, Institute of Genetics and Biotechnology, University of Warsaw, 02-106 Warsaw, Poland; 3 Institute of Biochemistry and Biophysics, Polish Academy of Sciences, 02-106 Warsaw, Poland

**Keywords:** photodynamic therapy, photosensitizer, reactive oxygen species, cytoskeleton, epithelial-mesenchymal transition, adhesion, survival pathway, APE1 inhibitor

## Abstract

Photodynamic therapy (PDT) is a low-invasive treatment method that can be used to treat VIN patients. A photosensitizer (PS) applied to a patient is activated with use of the appropriate wavelength of light, which in an oxygen environment leads to the formation of a reactive oxygen species (ROS) that destroys the tumor. However, cells can protect themselves against these cytotoxic products by increasing their antioxidant mechanisms and repair capacity. Changes in the cytoskeleton may also influence resistance to PDT. Our results revealed that PDT-resistant cells changed the amount of ROS. Cells resistant to PDT A-431 exhibited a decreased ROS level and showed higher viability after oxidizing agent treatment. Resistant Cal-39 cells exhibited a decreased O_2_^−^ level but increased other ROS. This provides protection from PDT but not from other oxidizing agents. Moreover, PDT leads to alterations in the cytoskeleton that may result in an epithelial-mesenchymal transition (EMT) or increased adhesion. Both EMT and cell adhesion may activate signaling pathways involved in survival. This means that resistance to PDT in vulvar cancer may be at least in part a result of changes in ROS level and alterations in the cytoskeleton.

## 1. Introduction

**Figure 1:**
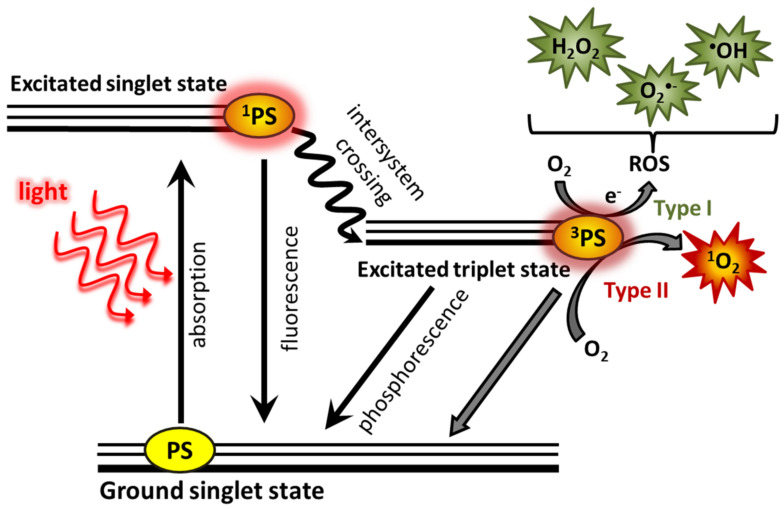
Mechanism of photosensitizer (PS) activation. Under illumination PS absorbs a photon and pass from a singlet ground state to a singlet excited state, and then, as a result of relaxation, to a triplet state. PS in the triplet state can interact with oxygen in a type I reaction to form reactive oxygen species (ROS) or in a type II reaction, leading to the formation of singlet oxygen.

Photodynamic therapy (PDT) is one of the less invasive treatment methods [1] causing the death of cancer cells [1,2]. A photosensitizer (PS) applied to the patient accumulates in tumor cells and its vascularity. PS is activated under the appropriate wavelength of light and oxygen, leading to the destruction of the tumor. The activation of PS takes place through the absorption of a photon, causing its transition to a singlet excited state with low stability. PS in the singlet state can return to the ground state through the energy loss by fluorescence. This phenomenon can be used for the detection and delineation of a tumor [1,3]. Alternatively, activated PS may lose some energy through a non-radiative transition to the excited triplet state, which has a longer lifetime than the excited singlet state [4]. Further loss of photosensitizer energy may occur through phosphorescence or as a result of collisions with other molecules in two types of reactions, type I and type II. These reactions lead to the formation of reactive forms (Figure 1) [1].

In the type I reaction, PS receive an electron or a proton which lead to the formation of radicals and ion radicals [1,4]. Typically, PS reacts with the electron supplying substrate [1] (e.g., NADPH, guanine in nucleic acids, and tryptophan and tyrosine in proteins) to form photosensitizer anion radical (PS^•−^) and biomolecule cation radical (biomolecule^•+^). In the oxygen environment, PS^•−^ transfers its additional electron to molecular oxygen, creating a superoxide radical anion (O_2_^•−^), which restores PS to the ground state [5].

Superoxide can oxidize small molecules and reacts with cellular radicals, releasing potentially cytotoxic products. In reaction with NO^•^, it forms a strong oxidant-peroxynitrite (ONOO^−^). This product reacts with CO_2_ and bicarbonates to form nitrosoperoxycarbonate (ONOOCO_2_^−^), a precursor of the carbonate radical anion (CO_3_^•−^), which may abstract electrons from tyrosine and tryptophan [5].

Furthermore, superoxide can lead to oxidation of [4Fe–4S] clusters located in proteins. These proteins are mainly dehydratases and Krebs cycle enzymes. The destruction of [4Fe–4S] clusters causes the inactivation of such proteins and affects the metabolic pathways in which they are involved. Moreover, iron released from [4Fe–4S] clusters bind to anionic molecules such as proteins, nucleic acids, lipids and other components of the cell membrane and is maintained in reduced form Fe^2+^ by cellular reductants. Upon encountering hydrogen peroxide (H_2_O_2_), it creates a strong oxidizing hydroxyl radical (HO^•^) via the Fenton reaction. HO^•^ can also be generated in the reaction of H_2_O_2_ reduction by the anion radical of the photosensitizer (PS^•−^). Due to its very high reactivity, HO^•^ damages objects that it encounters at the site of its creation [5].

When PS absorbs hydrogen, neutral radicals of the photosensitizer (PS^•^) and biomolecules (biomolecule^•^) are formed. In the presence of oxygen, hydrogen is transferred from the photosensitizer radical to molecular oxygen, leading to the formation of the hydroperoxide radical (HO_2_^•^) and the restoration of PS in its grand state [4].

In the type II reaction, PS in the triplet state transfers energy to molecular oxygen in the triplet state (^3^O_2_), creating the highly reactive singlet oxygen (^1^O_2_) [1], which is considered to be the basic component of PDT [3,5] that destroys the tumor and its vascularization [3]. Singlet oxygen interacts mainly with unsaturated compounds that contain double bonds [4], it can also react with neutral nucleophiles (such as sulfides and amines) and anions. Reactions with singlet oxygen lead to the formation of peroxides. The decomposition of peroxides generates radicals that can initiate various chemical reactions, producing biologically active products [5].

Most photosensitizers generate both singlet oxygen and radicals [5]. Both singlet oxygen and superoxide anion contribute to cytotoxicity, as they can induce damage to lipids, proteins, and nucleic acids [1]. Cells can protect themselves against this cytotoxicity by increasing antioxidant mechanisms and repair capacity [6,7]. However, unlike enzymes that protect against superoxide anion, living organisms have not developed enzymatic antioxidants to remove singlet oxygen [5].

On the other hand, reactive oxygen species (ROS) function as signaling molecules that may activate various signaling pathways [8,9,10]. Changes in the amount of these molecules may affect cell phenotype and response to treatment, as ROS are involved in cytoskeletal remodeling, proliferation, survival, migration, epithelial-mesenchymal transition (EMT), and drug resistance [10]. In our previous study, we showed that resistant CAL-39 cells become more elongated after seventh cycle of PDT. We also observed that they detached easier from the plate [6]. On the other hand, resistance to PDT did not induce any visible morphological changes in A-431 cells [6]. Nevertheless, they also change their adhesive properties, as they detached harder from the plate.

In this study we show that resistance to PDT may result from changes in ROS level in PDT-resistant cell lines. We also demonstrate that PDT affects cytoskeleton molecules in PDT-sensitive cells and leads to changes in cytoskeleton of PDT-resistant cells. These changes may play an important role in resistance to PDT as both EMT [10] and increased adhesion may activate survival pathways [7,11,12,13,14,15].

## 2. Results

### 2.1. Protein Profile of PDT-Sensitive and -Resistant Cells

**Figure 2:**
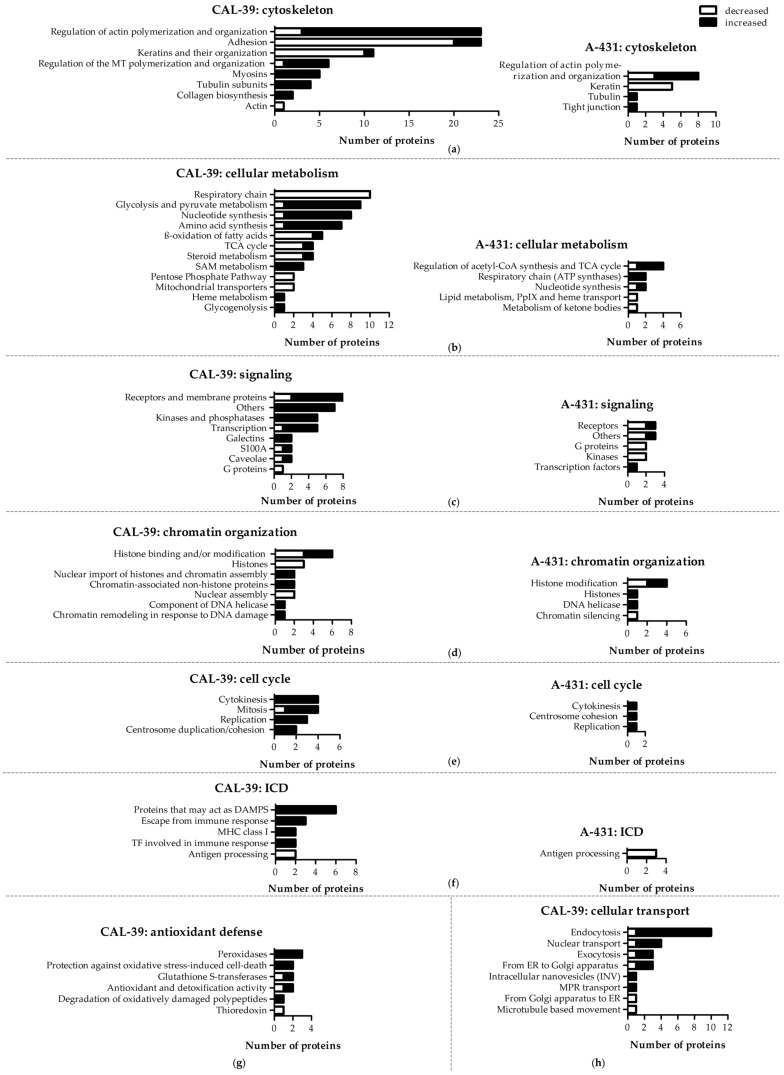
Changes in protein abundance in PDT-resistant cells as compared to the therapy-sensitive, parental cells. In both cell lines, this includes proteins involved in (**a**) organization and regulation of cytoskeleton, (**b**) cellular metabolism, (**c**) signaling, (**d**) chromatin organization, (**e**) cell cycle, (**f**) immunogenic cell death (ICD) and for the CAL-39 cell line, also (**g**) antioxidant defense and (**h**) cellular transport.

Proteomic profiles of parental and resistant cell lines were established by mass spectrometry (MS). Proteins with altered abundance in PDT-resistant cells compared to sensitive cells were grouped by processes in which they participate [6]. Here, we demonstrate more detailed analysis of the groups of proteins involved in formation and regulation of cytoskeleton and in cellular metabolism, as well as those involved in cellular signaling, chromatin organization, cell cycle, antioxidant defense, and cellular transport. We also demonstrate changes in protein level that may be involved in immunogenic cell death (Figure 2, Figure S1). The list of this proteins and their fold change are shown in Tables S1–S14 in Supplementary Materials. Additionally, RNA processing proteins that amount were change are also presented in Supplementary Materials (Tables S15 and S16). Other groups of proteins were shown in [6].

### 2.2. Cell Cycle

**Figure 3:**
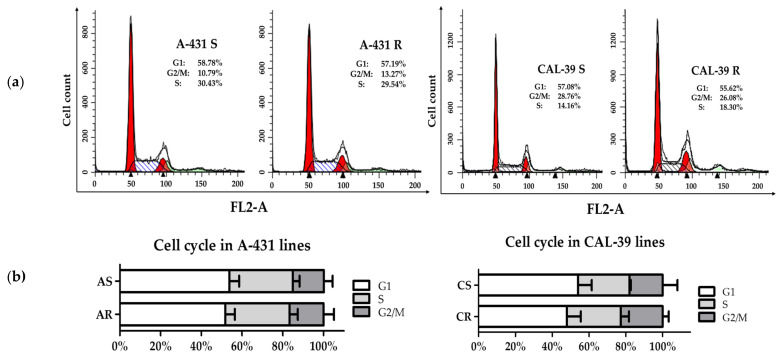
Cell cycle analysis. (**a**) Histograms of DNA content in cell. (**b**) Graphs of the percentage of cells in the G1, S, G2/M cycle phases.

Cancer cells tend to accumulate changes in the cell cycle. Accumulation of alterations leads to loss of homeostasis, affects the ability to respond to DNA damage, and can result in chemoresistance [16]. Resistant cells of both lines showed alterations in the number of proteins involved in cell cycle and chromatin organization (Figure 2, Supplementary Tables S7–S10). Therefore, cell cycle analysis was performed in parental and PDT-resistant cells. However, no significant differences were observed (Figure 3).

### 2.3. Assessment of Oxidative Stress

**Figure 4:**
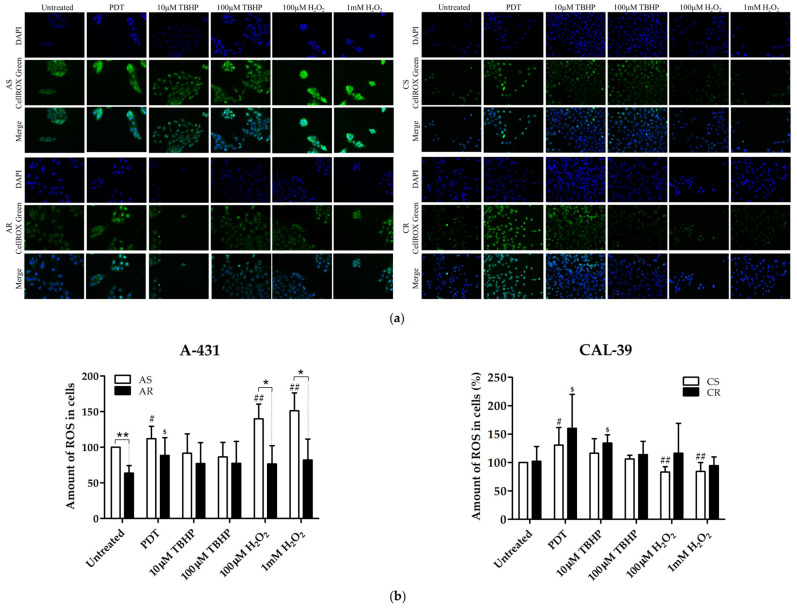
Assessment of oxidative stress. (**a**) Images of stained cells before and after treatment. Pictures were taken by the use of Olympus ScanR fluorescence microscope with 20× objective. (**b**) Graph with percentage values of mean fluorescence of untreated parental cells. Statistically significant differences between sensitive and resistant cells (* *p* ≤ 0.05, ** *p* ≤ 0.01) or between untreated and treated cells in sensitive (^#^ *p* ≤ 0.05, ^##^ *p* ≤ 0.01) or resistant (^$^ *p* ≤ 0.05) lines were calculated via Mann-Whitney test.

Oxidative stress was measured in untreated sensitive and resistant cells and after PDT, H_2_O_2_ or t-butyl hydroperoxide (TBHP). Resistant A-431 cells contained less ROS than the parental line. PDT increased ROS level in sensitive and resistant A-431 cell lines. H_2_O_2_ treatment significantly increased ROS level only in A-431 parental cells and THBP did not affect ROS amount in sensitive and resistant cells (Figure 4). These results suggest that resistance in A-431 cell line may be due to a reduction in ROS level. It is also possible that resistant cells have an increased ability to eliminate H_2_O_2_.

On the other hand, CAL-39 sensitive and resistant cells showed similar amounts of ROS. PDT increased ROS level in parental and resistant cells. Treatment with H_2_O_2_ led to the reduction in ROS levels in sensitive CAL-39 cells, while it did not cause significant changes in resistant cells. TBHP did not affect the amount of ROS in sensitive cells. In resistant CAL-39 cells, TBHP increased ROS content only at a lower concentration (10 μM) of TBHP. The lack of an increased ROS level in resistant cells after the application of a higher concentration (100 μM) TBHP may result from their increased mortality (Figure 4). These results suggest that resistance in CAL-39 cell line may result from higher tolerance of ROS induced by PDT. At the same time, these cells may be more susceptible to other oxidizing agents than their parental PDT-sensitive cells.

### 2.4. Cell Viability after H_2_O_2_ and TBHP Treatment

To verify if PDT resistance may be accompanied by an increased antioxidant capacity in the A-431 cell line or decreased ability to counteract some ROS in the CAL-39 cell line, the survival of PDT-sensitive and resistant cells after incubation with increasing concentrations of TBHP and H_2_O_2_ was compared.

**Figure 5:**
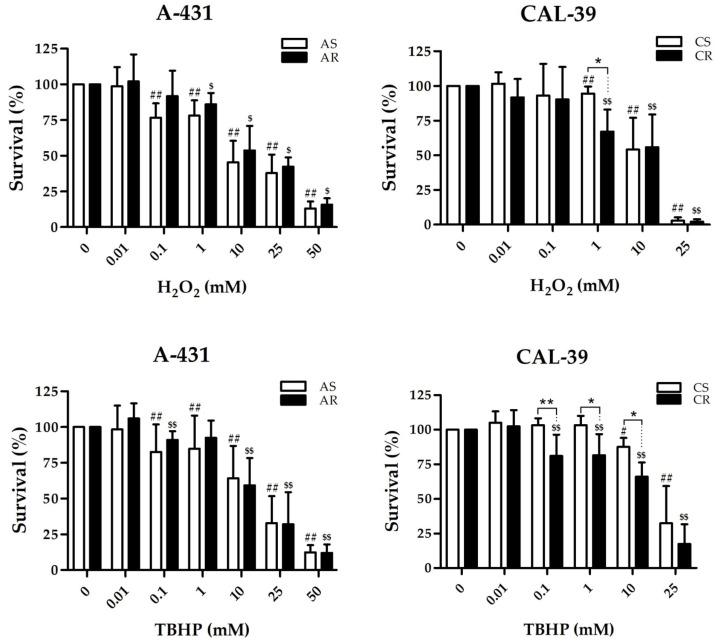
Cell survival after oxidative agents: H_2_O_2_ or TBHP. Statistically significant differences between sensitive and resistant cells (* *p* ≤ 0.05, ** *p* ≤ 0.01) or between untreated and treated cells in sensitive (^#^ *p* ≤ 0.05, ^##^ *p* ≤ 0.01) or resistant (^$^ *p* ≤ 0.05, ^$$^ *p* ≤ 0.05) lines were calculated via Mann-Whitney test.

No statistically significant differences were detected in the survival of sensitive and resistant A-431 cells after TBHP and H_2_O_2_ treatment. However, sensitive cells showed a reduction in survival after using a lower concentration (0.1 mM) of H_2_O_2_ than PDT-resistant cells (1 mM) compared to untreated cells (Figure 5). This may indicate that A-431 resistant cells have a better ability to eliminate H_2_O_2_ compared to sensitive cells.

Resistant CAL-39 cells showed lower survival after application of the tested oxidizing agents than sensitive cells. A decrease in the survival of both sensitive and resistant cells was observed after the application of 1 mM H_2_O_2_, however resistant CAL-39 cells showed a lower survival than their parental cells. Resistant CAL-39 cells also showed a significantly lower survival rate than sensitive cells after treatment with TBHP (0.1 mM, 1 mM, and 10 mM). Moreover, resistant cells showed decrease in survival after use a lower concentration (0.1 mM) of TBHP than sensitive cells (10 mM) (Figure 5). These results suggest that resistant to PDT CAL-39 cells are more sensitive to some oxidizing agents. Resistance in the CAL-39 cell line may be due to a reduced amount and/or an increased ability to eliminate particular ROS produced during PDT.

### 2.5. ROS Analysis in PDT-Sensitive and -Resistant Cells

Depending on the cell line and the oxidizing agent used, PDT-resistant cells may show different antioxidant capacity than their parental cells sensitive to this type of therapy. This may be associated with changes in particular ROS level. As PDT generates ^1^O_2_ and O_2_^•−^, which can lead to the formation of other reactive forms, including such strong oxidants as HO^•^ and ONOO^−^, we analyzed the amount of ^1^O_2_, O_2_^•−^ and HO^•^/ONOO^−^ in sensitive and resistant cell lines.

**Figure 6:**
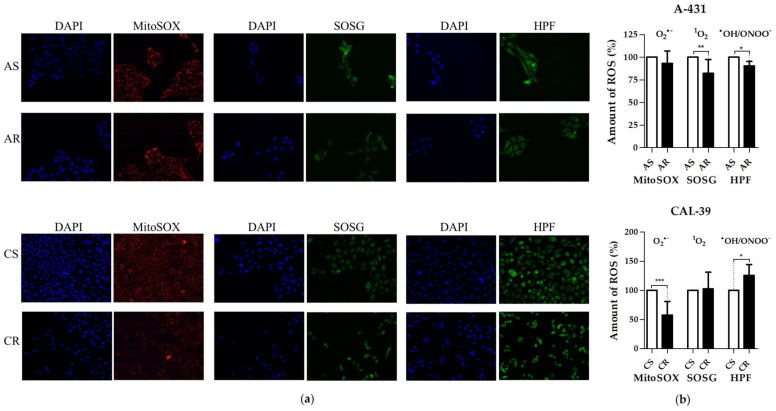
Analysis of ROS type. (**a**) Images of stained cells with Hoechst and MitoSOX, Singlet Oxygen Sensor Green (SOSG) or hydroxyphenyl fluorescein (HPF). Pictures were taken by the use of Olympus ScanR fluorescence microscope with 20× objective. (**b**) Graph with percentage values of mean fluorescence of untreated parental cells. Asterisks indicate the statistical significance of differences between sensitive and resistant cells calculated via Mann-Whitney test (* *p* ≤ 0.05, ** *p* ≤ 0.01, *** *p* ≤ 0.001).

A-431 resistant cells contained less ^1^O_2_ and HO^•^/ONOO^−^ than their parental cells, which may be one of the reasons of worse PDT response (Figure 6). The reduced amount of HO^•^/ONOO^−^ may result from the reduced amount of H_2_O_2_, as this would explain the decrease in survival after using higher concentration of this oxidizing agent than in the case of sensitive cells (Figure 5). The reduction in the amount of ^1^O_2_, HO^•^ and H_2_O_2_ is consistent with the results showing a decreased level of total ROS in these cells (Figure 4).

In contrast, PDT-resistant CAL-39 cells contained less O_2_^•−^ than their parental cells, but they also showed a higher amount of HO^•^/ONOO^−^ (Figure 6). The reduced amount of O_2_^•−^ in resistant CAL-39 cells may cause lower sensitivity to PDT. Changes in the level of O_2_^•−^ are in line with the results showing a decrease in the level of respiratory chain enzymes (Figure 2, Supplementary Table S3), as it is the main source of O_2_^•−^ [17]. Furthermore, an increased amount of HO^•^/ONOO^−^ with a decreased level of O_2_^•−^ in resistant cells suggests an elevated amount of H_2_O_2_ as ONOO^−^ is formed in the reaction of O_2_^•−^ with NO^•^ [5]. At the same time, an increased amount of HO^•^ and H_2_O_2_ with a decreased level of O_2_^•−^ in resistant cells may explain the lack of differences in the total amount of ROS between sensitive and resistant cells (Figure 4) and the lower survival of PDT-resistant cells after application of some oxidizing agents, including H_2_O_2_ (Figure 5). An alteration in the level of H_2_O_2_ may also explain changes in the amount of peroxidases (Figure 2, Supplementary Table S13). Their level could increase as a result of the adaptive response of cells to ensure redox homeostasis and protection against oxidative stress caused by changes in the amount of individual ROS.

### 2.6. Comparison of Mitochondria Amount

Mitochondria, apart from converting protoporphyrin IX (PpIX) into heme and its export to the cytoplasm for degradation/incorporation into hemoproteins, take part in other important cellular processes, including ATP production. Damage to mitochondria and inactivation of their enzymes may contribute to the rapid initiation of the apoptotic pathway. Several mitochondrial alterations, including their increased number, were reported in PDT-resistant clones [7]. Additionally, MS analysis (Figure 2) and previous studies of selected enzymes of heme metabolism [6] showed some changes in the number of mitochondrial enzymes in tested PDT-resistant cell lines of vulvar cancer. Therefore, in this work it was decided to compare the number of mitochondria in sensitive and resistant cells and in response to PDT.

**Figure 7:**
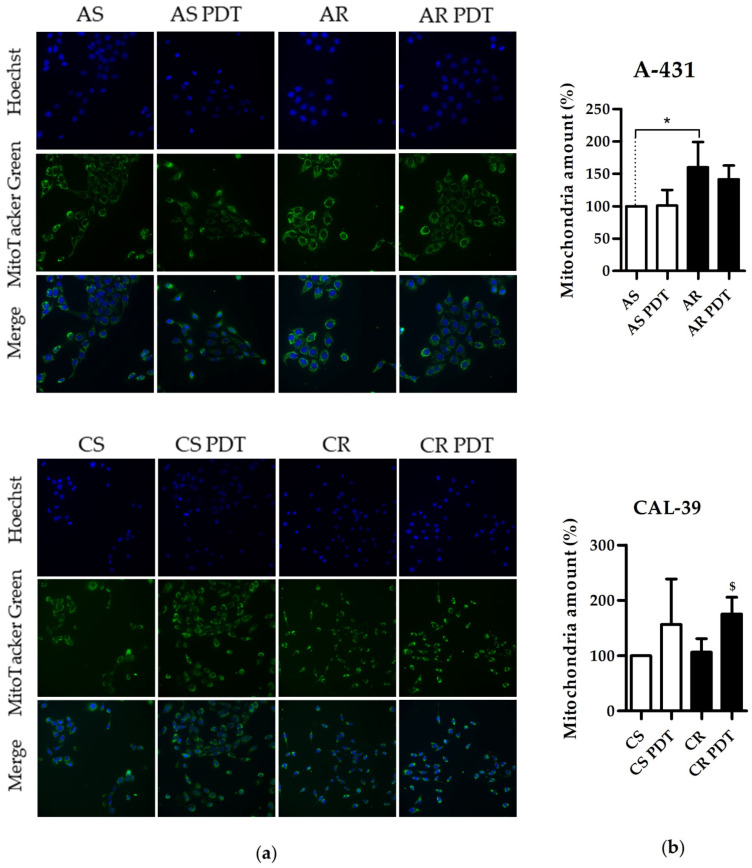
Comparison of the number of mitochondria. (**a**) Images of stained cells before and after treatment. Pictures were taken by the use of Olympus ScanR fluorescence microscope with 20× objective. (**b**) Graphs showing results as percentage of mean corrected total cell fluorescence (CTCF) value of untreated parental cells. Statistical significance between sensitive and resistant cells (* *p* ≤ 0.05) or between untreated and treated resistant cells (^$^ *p* ≤ 0.05) were calculated via Mann-Whitney test.

A-431 resistant cells had an increased number of mitochondria compared to sensitive cells (Figure 7). This result, together with the results of increased amounts of protoporphyrinogen oxidase (PPOX) and ferrochelatase (FECH) [6], suggests that resistant cells are characterized by an increased number of mitochondria and therefore an increased level of mitochondrial enzymes. An increased level of heme synthesis and an increased number of mitochondria may suggest an increase in the amount of other mitochondrial enzymes, including those of the respiratory chain. This, in turn, together with increased amount of ATP synthases presented in MS results (Figure 2, Supplementary Table S4) and increased respiration (Supplementary Figure S2), may suggest increased ATP synthesis in resistant cells.

No differences in the number of mitochondria were found between sensitive and resistant CAL-39 cells. However, PDT increased the number of mitochondria in CAL-39 resistant cells (Figure 7). This may suggest that mitochondria play an important role in counteracting the effects of PDT in CAL-39 resistant cells.

### 2.7. Comparison of Migration Rate

**Figure 8:**
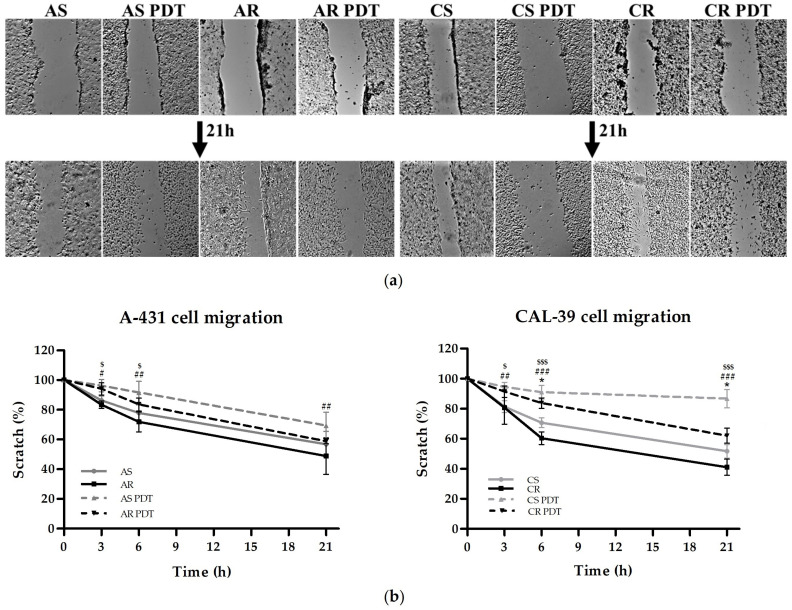
Assessment of migration rate. (**a**) Pictures of scratches at time 0 and after 21 h taken using an Olympus Xcellence fluorescence microscope with 4× objective. (**b**) Graphs showing the mean percentage of the scratch size. Statistical significance between groups were calculated in two-way ANOVA with Bonferroni post-test (differences between sensitive and resistant cells: * *p* ≤ 0.05; between untreated and treated cells in sensitive lines: ^#^ *p* ≤ 0.05, ^##^ *p* ≤ 0.01, ^###^ *p* ≤ 0.001 and in resistant lines: ^$^ *p* ≤ 0.05, ^$$$^ *p* ≤ 0.001).

Cell migration is a dynamic process related to the cytoskeleton. The main components of the cytoskeleton are actin microfilaments, microtubules and intermediate filaments [18]. ROS regulate various signaling pathways, including those responsible for cell migration [10]. Changes in ROS level acquired with resistance and ROS generated during PDT can affect the rate of cell migration. Therefore, the migration rates of sensitive and resistant cells and in response to PDT were compared. The results are presented as a percentage of the initial scratch size. The sensitive and resistant cells of the A-431 line show a similar migration rate. Resistant CAL-39 cells migrate faster than their parental cells. After PDT, resistant and sensitive cells of both lines had reduced migration abilities (Figure 8). These results suggest that PDT can cause oxidative damage to the cytoskeleton, leading to impaired cell migration capacity. Furthermore, the increased rate of migration, along with a change in the shape of resistant cells [6], numerous changes in the level of proteins that form and regulate the cytoskeleton (Figure 2, Supplementary Table S1), and an increased number of proteins involved in tumor progression (e.g., EGFR, CD44, STAT3, RELA in Supplementary Table S5) may suggest that resistance to PDT in CAL-39 cells increased at least partially through EMT.

### 2.8. Cytoskeletal Analysis

Changes in the structure of the cytoskeleton may lead not only to changes in the adhesion, shape, and rate of cell migration. They can also cause increased drug resistance [7,19,20] or a better response to oxidative stress [19,20]. Development of resistance in both cell lines was accompanied by changes in the number of proteins that regulate the actin and microtubule cytoskeleton. Additionally, the number of proteins involved in cell adhesion has changed (Figure 2, Supplementary Tables S1–S2). In CAL-39 cells, these changes may be related to EMT. Moreover, cytoskeleton is one of the cellular targets of PDT [7]. Therefore, we decided to test whether changes in the cytoskeleton may play a role in the acquisition of resistance to PDT. The amount of actin filaments (F-actin), β-actin and β-tubulin as well as E- and N-cadherin were compared between sensitive and resistant cells and before and after PDT. At the same time, the number of intermediate filaments of the cell nucleus-lamin A/C was also examined. Both of these lamins belong to the type A group. These proteins form a scaffold involved in various nuclear functions such as chromatin organization, replication, DNA repair, and transcription. Expression and stability of lamins change in response to oxidative stress [21]. Moreover, lamin A/C promotes base excision repair (BER) by enhancing the activity of APE1 and Polβ in PARylation associated manner, possibly by increasing the access and recruitment of these repair enzymes to the lesion site [22]. Type A lamins can also control the transcription and degradation of proteins involved in the repair of double strand break (DSB). The loss of type A lamins leads to the degradation of the 53BP1 protein essential for non-homologous end joining (NHEJ) and inhibits the expression of the BRCA1 and RAD51 proteins involved in homologous recombination (HR) [23,24].

**Figure 9:**
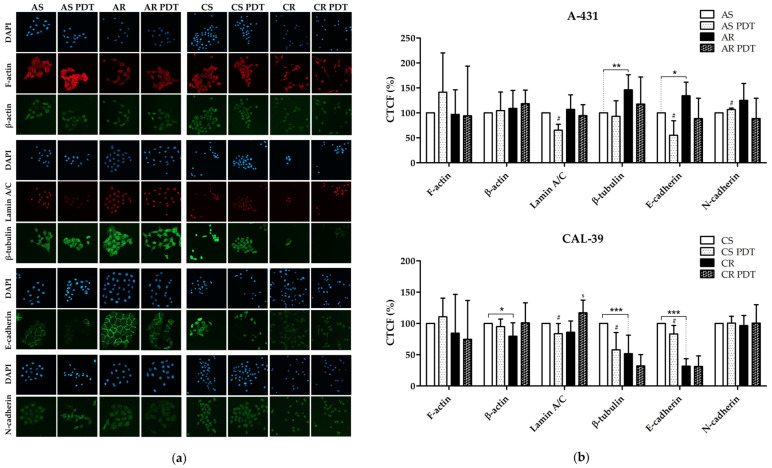
Analysis of the level of actin fibers, selected cytoskeleton proteins and lamin A/C; (**a**) Images of stained cells, before and after PDT treatment. Pictures were taken by the use of Zeiss LSM 800 confocal microscope with 20× objective. (**b**) Graph with percentage values of CTCF of untreated, parental cells. Statistically significant differences between sensitive and resistant cells (* *p* ≤ 0.05, ** *p* ≤ 0.01, *** *p* ≤ 0.001)) or between untreated and treated cells in sensitive lines: (^#^ *p* ≤ 0.05) or in resistant lines (^$^ *p* ≤ 0.05) were calculated via Mann-Whitney test.

Development of resistance to PDT in the A-431 line was accompanied by an increased β-tubulin and E-cadherin level. After PDT, statistically significant changes were only observed in the sensitive A-431 cells. There was a decrease in the level of lamin A/C and E-cadherin and an increase in the level of N-cadherin (Figure 9). These results suggest that elevated levels of β-tubulin and E-cadherin may lead to increased cell adhesion and promote PDT resistance. The decrease in the amount of E-cadherin after PDT in sensitive cells may result from PpIX accumulation in the plasma membrane [6]. The lack of a significant reduction in the amount of E-cadherin in resistant cells may be caused by lower accumulation of PpIX in these cells [6]. In sensitive cells as a result of PDT, ROS may promote adherens junction (AJ) disassembly and β-catenin/E-cadherin degradation [25]. ROS can also lower the level of E-cadherin by influencing the activity of transcription factors [10]. On the other hand, the increase in the level of N-cadherin may be a result of its stabilization by δ-catenin released from AJ [15]. The lack of changes in the level of β-tubulin after PDT in sensitive and resistant cells may suggest stabilization of microtubules and their protection against ROS.

The development of resistance in CAL-39 cells is accompanied by a decrease in levels of β-actin, β-tubulin and E-cadherin. PDT decreased the amount of β-tubulin, lamin A/C and E-cadherin in CAL-39 sensitive cells, and increased the level of lamin A/C in resistant cells (Figure 9). A significant reduction in E-cadherin levels in CAL-39 resistant cells may suggest EMT and confer resistance to the therapies. At the same time, changes in the amount of cytoskeleton proteins may explain changes in the shape, adhesion, and migration rate of resistant cells. Reduced levels of E-cadherin after PDT in sensitive cells may result, as in sensitive A-431 cells, from the localization of PpIX and/or activation of transcription factors that regulate its level. The lack of the effect of PDT on the amount of E-cadherin in resistant cells may result from different localization and lower accumulation of PpIX in these cells [6]. On the other hand, the decreased level of β-tubulin in resistant CAL-39 cells presented in Figure 9 compared to the MS results of increased number of β-tubulin subunits in resistant CAL-39 cells obtained from MS analysis (Figure 2, Supplementary Table S1) may indicate a change of β-tubulin isotypes, with a reduced amount of this protein. A significant reduction in the amount of β-tubulin after PDT only in sensitive cells may suggest protection of microtubules against ROS generated by PDT in resistant cells.

Changes in the amount of lamin A/C after PDT (Figure 9) also suggest that it may be significant for the response to therapy. Its increased expression in response to PDT-induced oxidative stress in resistant CAL-39 cells may lead to increased activity of DNA repair pathways after PDT and contribute to the resistance of these cells. Degradation of lamin A/C after PDT in both sensitive cell lines may suggest accumulation of oxidative DNA damage and DSB, which can lead to cell death. Decreased levels of lamin A/C after PDT may also indicate the initiation of apoptosis, as lamins are one of initial nuclear targets cleavage by caspases [21].

### 2.9. Influence of AR03, an APE1 Endonuclease Activity Inhibitor, on Cytoskeleton

APE1 is a dual-function enzyme that, through endonuclease activity, participates in DNA repair in BER pathway, and through redox activity acts as transcription factor. Nevertheless, APE1 through endonuclease activity may also affect the expression profile of many genes, and inhibition of APE1 endonuclease activity may contribute to a better response to PDT not only by inhibiting BER [6]. Since changes in the cytoskeleton, cell adhesion and amount of lamin A/C may affect cell resistance to PDT, the effect of AR03 on the amount of previously studied proteins and structures in PDT-resistant cells was examined.

The use of the APE1 inhibitor alone in resistant A-431 cells led to an increase in the amount of β-actin (40 μM), β-tubulin (20 and 40 μM), lamin A/C (40 μM) and N-cadherin (20 μM), and its combination with PDT increased the amount of β-tubulin and decreased the level of N-cadherin (40 μM).

**Figure 10:**
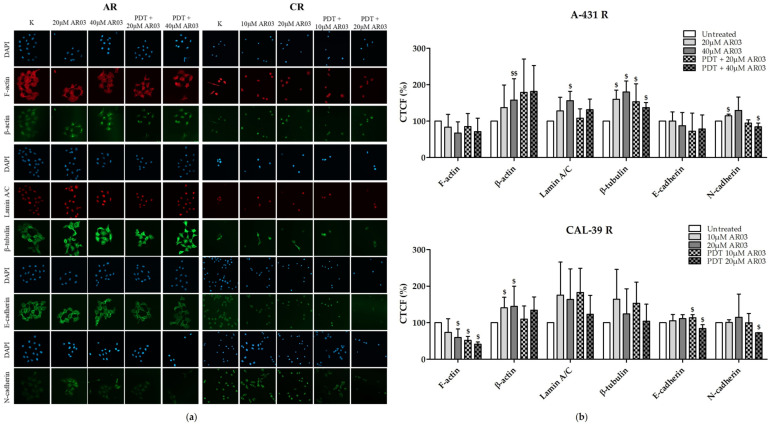
Effect of AR03 on cytoskeleton and lamin A/C. (**a**) Images of stained cells before treatment, after AR03 treatment, or after PDT combined with AR03 treatment. Pictures were taken by the use of Zeiss LSM 800 confocal microscope with 20× objective. (**b**) Graphs with percentage values CTCF of untreated cells, resistant to PDT. Statistically significant differences between resistant cells after treatment and untreated control were calculated via Mann–Whitney test (^$^ *p* ≤ 0.05, ^$$^ *p* ≤ 0.01).

In resistant CAL-39 cells, treatment with an AR03 inhibitor increased the amount of β-actin (10 and 20 µM) and decreased the level of F-actin (20 µM). Subjecting resistant CAL-39 cells to photodynamic therapy along with treatment with an APE1 inhibitor also led to the reduction in the amount of F-actin (10 and 20 µM). Additionally, combined therapy increased the amount of E-cadherin at a lower inhibitor concentration (10 μM), and a decrease in E- and N-cadherin at a higher concentration (20 μM) (Figure 10).

These results suggest the involvement of AP endonuclease activity in the regulation of the cytoskeleton. Inhibiting the activity of this endonuclease could therefore contribute to a greater sensitivity to PDT through disturbances in the cytoskeleton and cell adhesion. On the other hand, an increased amount of lamin A/C in response to AR03 inhibitor may suggest DNA repair attempts.

## 3. Discussion

**Figure 11:**
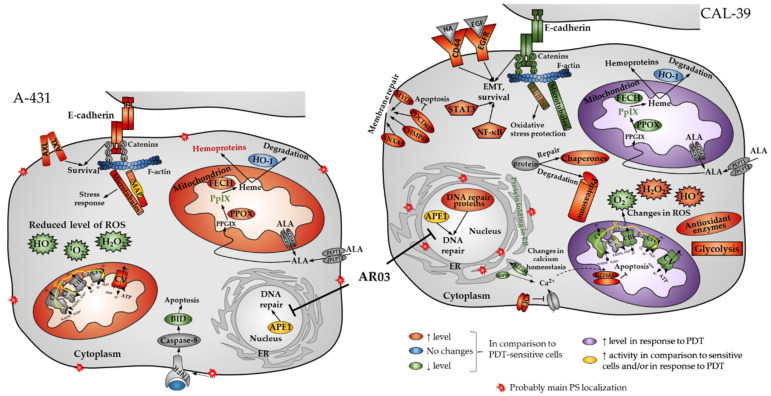
Mechanisms leading to resistance to photodynamic therapy (PDT) in vulvar cancer cells. Resistance to PDT vulvar cancer cells changed the amount of heme metabolism enzymes (PPOX, FECH) which led to decreased protoporphyrin IX (PpIX) levels in this cells [6]. Resistant cells also showed changes in the number of mitochondria and cellular metabolism, as well as ROS and antioxidant capacity. Changes in the cytoskeleton (E-cadherin, β-tubulin) that led to epithelial-mesenchymal transition (EMT) or increased adhesion can also contribute to resistance as a result of activation of survival pathways. Moreover, resistant cells increased the ability to repair/remove PDT-induced damage and could avoid cell death. Inhibition of APE1 DNA repair function effectively sensitizes cells to PDT [6]. The figure was prepared based on the results obtained by our group and described in this and previous [6] article and their Supplementary Materials.

PDT is a valuable method for treating precancerous conditions and early tumors. However, this kind of treatment is not always effective due to developing resistance to the treatment. Understanding the mechanisms of resistance may lead to the development of a more effective approach. Our research using two human vulvar cell lines (A-431 and CAL-39) revealed a number of changes that may contribute to the development of resistance to PDT in vulvar cancer (Figure 11).

In this work, we showed that resistant A-431 cells are characterized by an increased number of mitochondria in comparison to PDT-sensitive cells (Figure 7). The increased amount of these organelles in resistant A-431 cells leads to an increased amount of heme metabolism enzymes and a faster conversion of PpIX to heme [6]. A higher number of mitochondria may also increase the number of enzymes involved in aerobic respiration (Figure 2, Supplementary Table S4) and increased respiratory activity (Supplementary Figure S2). This may result in increased ATP level and cellular metabolism and reduce the effect of PDT.

On the other hand, resistant CAL-39 cells did not show changes in the number of mitochondria compared to parental PDT-sensitive cells but increased the number of mitochondria in response to PDT (Figure 7). Response to oxidative stress may increase the number of mitochondria to provide energy necessary for cell survival [26]. However, higher levels of the mitochondrial transporter SLC25A24 (Supplementary Table S13) may also indicate other mitochondrial function in response to PDT. This protein may assure the protection of cells from death induced by oxidative stress as well as high concentration of calcium ions [27]. In addition, CAL-39 resistant cells showed changes in the level of numerous mitochondrial proteins and other proteins involved in cellular metabolism. These changes may be associated with metabolic reprogramming. Resistant CAL-39 cells had an increased level of enzymes involved in glycolysis and reduced the amount of oxidative phosphorylation enzymes (Figure 2, Supplementary Table S3). This metabolic transition of cells from oxidative phosphorylation to glycolysis is called the Warburg effect and is considered a hallmark of cancer development and progression [28]. Although this process is much less efficient in terms of ATP production, it provides cancer cells with building blocks for macromolecule synthesis, the necessary redox conditions and the required energy [29]. This metabolic reprograming also minimizes ROS production in mitochondria [28]. By switching to aerobic glycolysis resistant CAL-39 cells could reduce the amount of O_2_^•−^ (Figure 6), as the respiratory chain is the main source of this ROS [17]. Superoxide and singlet oxygen are the main reactive oxygen species formed during PDT. Therefore, metabolic reprogramming, that leads to a significant reduction in O_2_^•−^ may have an impact on the efficacy of PDT. The lack of changes in the total amount of ROS in resistant CAL-39 cells (Figure 4) result from metabolic changes (Figure 2, Supplementary Table S3) leading to a decrease in O_2_^•−^ and an increased level of H_2_O_2_ and HO^•^. These changes may lead to increased resistance to PDT but also to increased sensitivity to other oxidizing agents (Figure 5). An increased pool of antioxidants may have also protective effect. These cells showed increased level of peroxidases, both glutathione- and thioredoxin-dependent, and glutathione S-transferase omega-1 (GSTO1) (Supplementary Table S13), an enzyme involved in the reduction of the oxidized form of ascorbic acid (ASC) – dehydroascorbate (DHA) [30,31] and detoxification of lipid peroxidation products by combining them with glutathione [17]. Elevated levels of these proteins and reduced levels of thioredoxin (Supplementary Table S13) may suggest a strong dependence of these cells on glutathione and ascorbate as antioxidant molecules.

These antioxidant molecules may also play a role in the acquisition of resistance in A-431 cells. As shown, resistant A-431 cells contain less ROS than sensitive cells (Figure 4). The decreased amount of ROS in resistant A-431 cells is caused by the lower content of ^1^O_2_, H_2_O_2_ and HO^•^, which results in reduced effectiveness of PDT, but also in some other oxidizing agents, such as H_2_O_2_ (Figure 5). Taking into consideration that there are no enzymatic mechanisms to eliminate ^1^O_2_ [5], the decreased level of this ROS (Figure 6) in resistant A-431 cells may be caused by increase in the amount of such molecules as bilirubin, ascorbate (ASC), tocopherols and carotenoids, which are capable of quenching various ROS, including singlet oxygen [17]. Resistant A-431 cells may produce more bilirubin as a result of changes in heme metabolism and heme oxygenase activity. Alternatively, resistant cells could increase the production of antioxidant hemoproteins, such as, for example, catalase [6]. Moreover, taking into account greater tolerance to H_2_O_2_ and a decreased level of HO^•^ in resistant A-431 cells than in the parental line, an increased amount of glutathione may promote the restoration of damaged cellular components, proteins and lipids also in this resistant cell line.

Glutathione is an essential component of antioxidant defense, involved in the reduction of organic peroxides, hydrogen peroxide, and free radicals, both HO^•^ and organic radicals. The quenching reaction is accelerated by glutathione peroxidases [17]. Due to the high importance of the glutathione system in the elimination of various ROS, the inhibition of glutathione synthesis could have a beneficial effect on the effectiveness of PDT. The increased level of reduced glutathione [7] and the number of enzymes utilizing it were previously associated with protection against PDT in various cell lines [7,32]. Moreover, it was shown that inhibition of glutathione synthesis influenced the cytotoxicity of PDT in the MCF-7 line by increasing ROS levels and led to apoptosis [33].

On the other hand, ascorbate, despite its strong reducing properties against various ROS [17], may show a pro-oxidative effect at high concentration. Preclinical and clinical studies suggest that it can be administered safely to patients in high doses and improve the anticancer effect of chemo- and radiotherapy [34]. Considering the importance of nonenzymatic antioxidants in the protection of cells against ROS generated during PDT, it can be assumed that additional administration of ascorbate, leading to changes in its antioxidant properties to pro-oxidative properties, may also improve the effectiveness of this therapy.

Nevertheless, changes in ROS level may also affect PDT efficiency by influence on signaling pathways. ROS are involved in the activation of various signaling pathways that can lead to changes in proliferation, metabolism, angiogenesis [9], adhesion, migration, invasion, and EMT [10]. The main ROS involved in redox signaling is H_2_O_2_, the amount of which was changed in resistant cells of both lines. H_2_O_2_ is produced in response to EGF [8], leads to inactivation of inhibitory phosphatases of PI3K and AKT [8,9,35], which may result in increased activity of the EGFR/PI3K/AKT pathway, increased proliferation and survival. The amount of EGFR (Supplementary Tables S5 and S6) was changed in the same way as H_2_O_2_ in resistant cell lines. An increased amount of this receptor was observed in resistant CAL-39 cells. Thus, the EGFR/PI3K/AKT pathway may play a role in the development of resistance in CAL-39 cells and be one of the reasons for the observed changes in cellular metabolism (Figure 2, Supplementary Table S3) as the activation of AKT may promote aerobic glycolysis by affecting glucose uptake and the glycolysis pathway [35]. Moreover, the PI3K/AKT pathway may also facilitate protein synthesis, promote EMT by activating the NF-κB pathway and increase cell migration [10]. Resistant CAL-39 cells increased the level of proteins involved in synthesis of proteins [6] and one of the NF-κB subunits (RELA, Supplementary Table S5), showed an increased rate of migration (Figure 8), as well as numerous changes in the cytoskeleton (Figure 2, Figure 9, Supplementary Table S1) that could confirm the epithelial-mesenchymal transition. EMT is an adaptive process that can confer cancer cells a stem-like phenotype to survive lethal stimuli and develop drug resistance [10]. CD44 is one of the key markers of cancer stem cells [10,36]. Its amount also increased in resistant CAL-39 cells (Supplementary Table S5).

However, both resistant human vulvar cancer cell lines showed changes in cytoskeleton. Moreover, we demonstrated that PDT also affect cytoskeleton structures (Figure 9). In contrast to resistant CAL-39 cells, which showed a decrease in the level of E-cadherin (Figure 9, Supplementary Table S1), catenins (Supplementary Table S1) and many other cell adhesion molecules (Supplementary Table S1), resistant A-431 cells increased the level of E-cadherin (Figure 9) and tight junction protein (Supplementary Table S2). As a result of PDT, the level of E-cadherin decreased in both sensitive cell lines (Figure 9). As mentioned above, changes in resistant CAL-39 cells may be associated with EMT and resistance to treatment. However, also increased amount of E-cadherin in resistant A-431 cells may lead to a poorer response to the therapy. The intercellular interactions created by E-cadherin were previously linked with protection of cancer cells against chemotherapy [11,12,13,14,15]. For this reason, the degradation of E-cadherin in sensitive cells of both lines due to ROS action after PDT may contribute to their death, and the increased amount of E-cadherin in resistant A-431 cells may lead to a weaker response to treatment.

Changes in the microtubule cytoskeleton may also promote resistance to PDT. Resistant A-431 cells showed an increased amount of β-tubulin, and resistant CAL-39 cells decreased the level of this protein (Figure 9), with an increase in some isotypes (Supplementary Table S1). Microtubules play a role in cell adhesion [37,38], which may promote resistance to various therapies by influencing antiapoptotic pathways [7]. Additionally, changes in microtubule stability, tubulin isotypes expression and its post-translational modifications have been correlated with poor prognosis and resistance to chemotherapy [20]. The lack of changes in β-tubulin level after PDT in both sensitive and resistant A-431 cells may suggest stabilization of microtubules and their protection against ROS. MAP proteins protect microtubules against depolymerization in response to oxidative stress. Their interactions with tubulin also affect sensitivity to chemotherapy [20]. Thus, an increase in microtubule level in resistant A-431 cells may contribute to resistance by influencing cell adhesion, and MAP proteins likely favor PDT resistance by ensuring high microtubule stability. In turn, in the CAL-39 cell line, PDT causes a significant decrease in β-tubulin level only in sensitive cells (Figure 9). ROS generated during PDT can depolymerize microtubules unbound with MAP proteins and damage β-tubulin. However, reduced susceptibility to microtubule cytoskeleton damage may result from changes in β-tubulin isotypes. Specific β-tubulin isotypes can provide protection against oxidative stress. These include tubulins of βIII, βV and βVI classes, which, acting as redox switches, can alter the response to oxidative stress. These proteins contain cysteine at the ser/ala124 position and serine instead of cys239. ROS, by oxidizing β-tubulin in cys239, inhibits the microtubule assembly and stabilization [20] and damages the cytoskeleton by reducing the amount of microtubules and tubulin [10]. Resistant CAL-39 cells showed, among others, increased level of βV-tubulin (TUBB6, Supplementary Table S1). On the other hand, the β-tubulin isotypes susceptible to oxidation in CAL-39 sensitive cells may lead to significant destruction of microtubules after PDT (Figure 9) and contribute to their death. Moreover, destruction or stabilization of the microtubule network may block migration [38], which is consistent with decreased migration rate in response to PDT in all tested cells (Figure 8). In addition, tubulins and microtubule-related proteins may also play a role in the response to cellular stress, thus ensuring increased survival of cancer cells. Microtubules can participate in signal transduction in response to cellular stress, influence the activity of MAPK pathways and the stress response dependent on TP53. The microtubule network probably may also be involved in the regulation of apoptosis [20].

In this study, we also analyzed whether the APE1 endonuclease inhibitor, which effectively sensitized vulvar cancer cells to PDT [6], may affect the cytoskeleton. Resistant A-431 cells showed higher levels of β-tubulin, and resistant CAL-39 cells showed lower levels of F-actin in response to PDT combined with AR03 treatment (Figure 10). This may indicate that inhibition of APE1 promotes assembly and stabilization of microtubules in resistant A-431 cells and depolymerization of F-actin in resistant CAL-39 cells. Changes in the dynamics of actin or microtubules can lead to cell death. Agents that inhibit actin polymerization and destabilize existing F-actin [39,40] or promote microtubule assembly and stabilization [20] have been linked to induction of apoptosis. The APE1 inhibitor in combination with PDT also decreased the amount of cadherins in resistant cells (Figure 10). The reduction in the amount of cadherins may lead to the inhibition of their protective effect on the survival of cancer cells. As mentioned above, E-cadherin-mediated cell adhesion may promote cancer cell survival. However, N-cadherin dependent adhesion also was associated with inhibition of apoptosis [15]. Therefore, APE1 inhibition may improve the effect of PDT not only by reduction of DNA repair efficacy, but also by affecting other processes, such as those that lead to cytoskeletal disruption (Figure 10) or changes in heme metabolism pathway [6].

## 4. Materials and Methods

### 4.1. Cell Cultures

Two human vulvar squamous cancer cell lines were used as a VIN model. A-431 was purchased from ATCC and CAL-39 from DSMZ. This cell lines are described as sensitive ones. Resistant cells were isolated by subjecting them to repeated PDT cycles as described in [6]. Sensitive and resistant A-431 cell lines were cultured in RPMI-1640 medium (Gibco by Thermo Fisher Scientific, Waltham, MA, USA) supplemented with 10% fetal bovine serum (FBS; Gibco) and 1% penicillin–streptomycin antibiotic solution (Pen-Strep; HyClone by Thermo Fisher Scientific, Waltham, MA, USA). Sensitive and resistant CAL-39 cells were maintained in Dulbecco’s modified Eagle’s medium (DMEM; HyClone) with 20% FBS, 2 mM L-glutamine (Gibco), 1 mM sodium pyruvate (Gibco), 0.5 nM hydrocortisone (Sigma-Aldrich, Saint Louis, MO, USA), 1 μg/100 mL EGF (Gibco), and 1% Pen-Strep. All cell lines were cultured at 37 °C in a humidified incubator with 5% CO_2_ and atmospheric oxygen concentration.

### 4.2. PDT-Treatment

Cells were incubated for 3 h in serum-free medium with 0.6 mM 5-aminolevulinic acid (5-ALA; precursor of actual PS - PpIX) (Sigma-Aldrich). Then, cells were exposed to red light (630 nm wavelength, 187 W/m^2^ power density) emitted by LED lamp with a dose describe in each experiment.

### 4.3. Mass Spectrometry Analysis

Protein extraction, digestion, measurement, and analysis were performed as described in [6]. Proteins with *t*-test value ≤ 0.05 and fold change of resistance to sensitive cell ≥ 1.5 or ≤ 0.67 were taken for further analysis. These include 85 proteins in line A-431 and 390 in CAL-39, which were analyzed with KEGG Mapper. The proteins’ functions were checked in the Uniport/Genecards databases (September 2019) and, if necessary, in additional publications.

### 4.4. Cell Cycle Analysis

Cells were seeded on 10 cm dishes. After reaching 70–80% confluency, 10^6^ cells were collected and centrifuged (4 min, 400 × g at room temperature). Cells were washed with PBS, resuspended in PBS, fixed overnight in cold 70% ethanol (4 °C). The next day, cells were centrifuged (7 min, 600 × g at 4 °C), washed with PBS and resuspended in PBS containing 50 µg/mL propidium iodide (PI) and 50 µg/mL RNase A (Sigma-Aldrich). Samples were incubated with the staining solution for 30 min in the dark on a shaker (50 rpm) at 26 °C and then analyzed on a BD FACS Calibur flow cytometer (BD Biosciences, NJ, USA) with the detector set to FL2A channel to detect excited PI. Data were collected in BD CellQuest Pro software (BD Biosciences) and presented as histograms showing DNA content of the cell nuclei. Further analysis was performed using ModFit LT software (BD Biosciences). The experiment was carried out in at least triplicate using independent cell cultures.

### 4.5. Measurement of Oxidative Stress Using CellROX Green Reagent

Cells were seeded in 24-well plates at a density of 2 × 10^5^ per well for A-431 cell lines or 1.5 × 10^5^ per well for CAL-39 cell lines. Cells of each line were subjected to PDT (with light dose of 11.2 J/cm^2^ for CAL-39 cells or 16.8 J/cm^2^ for A-431 cells), treated with H_2_O_2_ (100 μM and 1 mM) or TBHP (10 μM and 100 μM). Untreated cells were used as a control. After treatment, in all wells, the medium was changed to that containing 5 µM CellROX Green reagent (Thermo Fisher Scientific) and cells were incubated for 30 min at 37 °C. Afterwards, cells were washed three times with PBS, fixed in 3.7% formaldehyde (in PBS), washed twice with PBS and stained with DAPI (Vector Laboratories, Burlingame, CA) in PBS (1: 1000) for 30 min. At least 10 random pictures were taken on each well using ScanR fluorescence microscope (Olympus, Tokyo, Japan) with UPlanSApo 20.0× NA 0.75. Images were processed and analyzed with ImageJ software (National Institutes of Health, Bethesda, MD, USA). The results are presented as a percentage of the mean fluorescence intensity of the parental untreated cells. The experiment was carried out at least in triplicate using independent cell cultures.

### 4.6. Cell Viability Assay

Cells were seeded 24 h before the experiment at a density of 6 × 10^3^ cells per well. The next day, cells were incubated for 1.5 h with TBHP or H_2_O_2_ at concentrations: 10 μM-50 mM for A-431 cells and 10 μM-25 mM for CAL-39 in serum-free medium. Control cells were incubated in serum-free medium. After treatment, cells were incubated for 4 h with 10% alamarBlue Cell Viability Reagent (Invitrogen, Thermo Fisher Scientific, Waltham, MA, USA) in complete medium at 37 °C in a cell culture incubator. Fluorescence intensity was measured using a DTX 880 Multimode Detector (Beckman-Coulter; Brea, CA, USA) at 540 nm excitation and 590 nm emission wavelengths. The fluorescence ratio of the tested groups to the untreated control was calculated and presented as a percentage value of the control. Cell viability assay was performed at least three times with independent cell cultures.

### 4.7. Analysis of the Type of ROS

Cells were seeded in black 24-well plates (A-431 cell lines and sensitive CAL-39 cells at a density of 1,5 × 10^4^ per well and resistant CAL-39 cells at 4 × 10^4^ per well). After 3 days, cells were incubated: 1) 3 h with 20 μM Singlet Oxygen Sensor Green (Invitrogen), which in the presence of ^1^O_2_ emits green fluorescence, 2) 10 min with 5 μM MitoSOX Red (Invitrogen), which as a result of oxidation by O_2_^•−^ produces red fluorescence, or 3) 3 h with 20 μM hydroxyphenyl fluorescein (Invitrogen), which after oxidation by HO^•^ or ONOO^−^ shows green fluorescence. In addition, cell nuclei were stained for 20 min with 5 µg/mL Hoechst 33342 (Invitrogen) in all variants of the experiment. Twenty-five random pictures were taken for each well using ScanR fluorescence microscope (Olympus) with UPlanSApo 20.0× NA 0.75. The analysis was performed automatically by related software. At least three independent experiments were conducted.

### 4.8. Comparison of Mitochondria Amount

Cells were seeded in black 96-well plates. The next day, cells were subjected to PDT with a light dose of 1.1 J/cm^2^ for CAL-39 cell lines and 3.4 J/cm^2^ for A-431 cell lines. Untreated cells were used as controls. Then, in all wells, medium was changed and cells were incubated for 30 min in serum-free medium containing 100 nM MitoTacker Green (Invitrogen), 2.5 μg/mL Hoechst 33342, after which cells were washed with PBS and flooded with serum-free medium. Pictures were taken using ScanR fluorescence microscope (Olympus) with UPlanSApo 20.0× NA 0.75. The mean value of corrected total cell fluorescence (CTCF) was calculated for each variant of the experiment using ImageJ. The experiment was performed at least in triplicate using independent cell cultures, and the results were presented as the percentage of mean CTCF of untreated parental cells.

### 4.9. Scratch Assay

The rate of migration was measured using the scratch assay. Cells were seeded in 24-well plates at the density of 3 × 10^5^ cells per well for A-431 cells, 4.5 × 10^5^ for sensitive CAL-39 cells and 5.5 × 10^5^ for resistant CAL-39 cells. After reaching 95–100% confluence, PDT was performed with light dose of 1.1 J/cm^2^ for CAL-39 cells and 3.4 J/cm^2^ for A-431 cells. Untreated cells were used as controls. The medium was removed and in each well scratch was made. Cells were rinsed with PBS and flooded with serum free medium. Pictures were taken at 0, 3, 6 and 21 h after scratch was performed using the Xcellence fluorescence microscope (Olympus) with the UplanFL N 4× objective with phase contrast (Olympus). Scratches were measured in 10 places for each image in ImageJ and converted to percent of the size of the scratch relative to time 0. The experiment was carried out at least in triplicate using independent cell cultures.

### 4.10. Immunofluorescence

The experiment was performed as described in [6]. Cells seeded in 24-well plates were cultured until 70% confluence was reached and treated with (1) PDT, (2) APE1 inhibitor, or (3) PDT with APE1 inhibitor. Untreated cells were used as a control. Afterwards, cells were washed 3 times with PBS, fixed with 3.7% paraformaldehyde in PBS, permeabilized with 0.2% Triton X-100 in PBS and again washed 3 times with PBS. Subsequently, cells were blocked in SuperBlock (PBS) (Thermo Scientific) with 0.025% Triton X-100, washed 5 times with PBS containing 0.5% BSA, and incubated overnight with primary antibodies (Table 1). The next day, cells were washed 5 times with PBS containing 0.5% BSA and incubated for 1 h at room temperature in the dark with secondary antibodies (Table 1) or phalloidin-iFluor 647 (Abcam) diluted 1:1000 in PBS containing 0.5% BSA.

### 4.11. Statistical analysis

Data presented in Figures are means ± SD of at least three independent experiments. Statistical significance of differences between groups was estimated by two-tailed Mann-Whitney test or two-way analysis of variance (two-way ANOVA) followed by Bonferroni post-tests. Differences were considered significant if the *p*-value was ≤ 0.05. Data was analyzed and visualized using GraphPad Prism 5.03 (GraphPad Software)

Samples were washed with limited access to light: 5 times with PBS containing 0.5% BSA and 3 times with PBS and then cell nuclei were stained with Fluoroshield Mounting Medium With DAPI (Abcam). Six random pictures of each well were taken using Zeiss LSM 800 confocal microscope (20× objective). The images were analyzed with ImageJ software and CTCF was calculated. This experiment was carried out at least three times with an independent cell culture. Results showing changes that occurred with development of resistance and in response to PDT in sensitive and resistant cells are presented as percentage values of sensitive cell fluorescence. Results of APE1 inhibitor treatment alone and in combination with PDT are presented as percentage values of untreated resistant cell fluorescence.

## 5. Conclusions

In our studies, the resistance to PDT in vulvar cancer cell lines developed as a result of changes in the amount of heme metabolism enzymes (PPOX, FECH) that led to decreased amount of PpIX [6], changes in the number of mitochondria and cellular metabolism, as well as ROS and antioxidant capacity. Changes in the cytoskeleton (E-cadherin, β-tubulin) can also contribute to resistance as a result of activation of survival pathways. However, obtained results mostly differ between the two tested cell lines, which suggests that the resistance of vulvar cancer cells to PDT is not influenced by the same mechanisms. Nevertheless, inhibition of APE1 DNA repair function effectively sensitizes cells to PDT [6].

## Figures and Tables

**Table 1:** Antibodies used in the immunofluorescence experiment. Antibodies were purchased from Abcam (Cambridge, England) Santa Cruz Biotechnology, (Dallas, TX) or Invitrogen.

Primary Antibodies	Company	Dilution	Secondary Antibody
Anti-β-actin	Santa Cruz	1:1000	Goat anti-mouse antibody, Alexa Fluor 488 (Abcam)
Anti-β-tubulin	Santa Cruz	1:1000	
Anti-N-cadherin	Invitrogen	1:500	Goat anti-rabbit antibody, Alexa Fluor 594 (Abcam)
Anti-E-cadherin	Abcam	1:500	
Anti-lamin A/C	Abcam	1:500	

## Data Availability

Not applicable.

## Supplementary Materials

The following supporting information can be downloaded at: https://www.mdpi.com/article/10.3390/ijms232314689/s1, Table S1: Changes in the level of proteins involved in formation and regulation of cytoskeleton in resistant CAL-39 cells; Table S2: Changes in the level of proteins involved in formation and regulation of cytoskeleton in resistant A-431 cells; Table S3: Changes in the level of proteins associated with metabolic processes in resistant CAL-39 cells; Table S4: Changes in the level of proteins associated with metabolic processes in resistant A-431 cells; Table S5: Changes in the level of proteins involved in cellular signaling in resistant CAL-39 cells; Table S6: Changes in the level of proteins involved in cellular signaling in resistant A-431 cells; Table S7: Changes in the level of proteins involved in chromatin regulation in resistant CAL-39 cells; Table S8: Changes in the level of proteins involved in chromatin regulation in resistant A-431 cells; Table S9: Changes in the level of proteins involved in cell cycle in resistant CAL-39 cells; Table S10: Changes in the level of proteins involved in cell cycle in resistant A-431 cells; Table S11: Changes in the level of proteins that may be involved in immunogenic cell death in resistant CAL-39 cells; Table S12: Changes in the level of proteins involved in antigen processing in resistant A-431 cells; Table S13: Changes in the level of proteins involved in antioxidant defense in resistant CAL-39 cells; Table S14: Changes in the level of proteins involved in vesicular transport in resistant CAL-39 cells; Table S15: Changes in the level of proteins involved in RNA processing in resistant CAL-39 cells; Table S16: Changes in the level of proteins involved in RNA processing in resistant A-431 cells; Figure S1: Immunogenic cell death and resistance to PDT; Figure S2: Mitochondrial respiration.
