# Supplementary material for: Possible Mechanisms of Resistance Development to Photodynamic Therapy (PDT) In Vulvar Cancer Cells
Source: Int J Mol Sci. 2022 Nov 24;23(23):14689. doi: 10.3390/ijms232314689 (PMC9741432; doi:10.3390/ijms232314689)
Supplement: Supplementary file 1 [file ijms-23-14689-s001.zip › ijms-1993529-supplementary.pdf]

Possible Mechanisms of Resistance Development to Photodynamic Therapy (PDT) In Vulvar Cancer Cells

Beata Joanna Mossakowska [1,*], Anna Fabisiewicz [1], Barbara Tudek [2,3,†] and Janusz Aleksander Siedlecki [1]

[1] Department of Molecular and Translational Oncology, Maria Skłodowska-Curie National Research Institute of Oncology, 02-781 Warsaw, Poland
[2] Faculty of Biology, Institute of Genetics and Biotechnology, University of Warsaw, 02-106 Warsaw, Poland
[3] Institute of Biochemistry and Biophysics, Polish Academy of Sciences, 02-106 Warsaw, Poland
* Correspondence: beata.mossakowska@pib-nio.pl
† Deceased.

SUPLEMENTARY MATERIALS

Proteome comparison of PDT resistant cell lines to their parental lines

Despite the large differences between the two PDT-resistant vulvar cancer cell lines, they also showed some common features. Both lines had elevated levels of LASP1, ATIC, ST13, CDV3 proteins, and decreased HM13 proteins. These changes may be involved in the development of resistance to PDT.

LASP1, the actin-binding cytoskeleton protein, which localizes in cellular protrusions dedicated to migration and predominantly is involved in the reorganization of cytoskeleton during cellular motility. In focal adhesions (FA), it regulates anchoring to the ECM [1] and probably participates in mediating survival signals from these structures [2]. LASP1 plays a role in vesicle secretion, matrix metalloproteinase expression, transcription regulation, and activation of survival and proliferation pathways in different types of cancer. It may influence the expression of AP-1 dependent genes and positively regulate the PI3K/AKT pathway. Furthermore, after serine 146, LASP1 can be translocated along with ZO-2 to the cell nucleus, where it contributes to SNAIL-dependent epigenetic silencing of E-cadherin expression. Additionally, LASP-1 influence on the expression of several others proteins [2] and the activity of signaling pathways may suggest its function in EMT. In colorectal cancer cells, LASP1 led to EMT by activation of MAPK, PI3K/AKT and Smad. In contrast, in non-small cell lung cancer, LASP1 promoted proliferation and invasion through FAK-AKT regulation [3]. Due to the possible role of LASP1 in promoting cell survival and/or EMT, this protein could be a potential target for combined therapy with PDT.

ATIC is an enzyme involved in de novo purine biosynthesis pathway. The appropriate level of purines in mammalian cells is maintained by the coordinated action of their de novo biosynthetic and recovery pathways. Normal cells appear to preferentially use recovery for purine synthesis, while cancer cells prefer the de novo pathway to support increased RNA production and DNA replication. Inhibition of ATIC may impair cell proliferation and migration and lead to apoptosis [4]. Thus, inhibition of ATIC could positively influence the efficacy of PDT, and the differences in purine metabolism between healthy and neoplastic cells could ensure lack of increase in toxicity of such therapy.

ST13 is the co-chaperone of HSP70 family proteins. This protein supports the chaperone function of HSP70 in both protein folding and repair process and in controlling the activity of regulatory proteins

such as steroid receptors and regulators of proliferation or apoptosis [5]. Therefore, along with HSP70, it may probably contribute to the reduction in the PDT effect.

CDV3 is a protein of unknown function, that likely plays a role in cell proliferation [6]. CDV3 is also correlated with tumorigenesis and may affect some biological processes in cancer [7].

HM13 is involved in the formation of HLA-E surface epitopes from MHC-I signal peptides. HLA-E expression on the surface of tumor cells can lead to immunosuppression [8].

Both cell lines could also activate the PI3K/AKT, MEK/ERK, NF-κB or STAT3 pathways. In resistant CAL-39 cells, among others, increased receptors may be responsible for their activation [9-16]. In resistant A-431 cells, an increased amount of the AXL receptor may lead to activation of the above-mentioned pathways [17].

Changes in the level of proteins involved in formation and regulation of cytoskeleton

Table S1. Changes in the level of proteins involved in formation and regulation of cytoskeleton in resistant CAL-39 cells.

		Gen	T-Test	CR:CS
**Regulation of actin polymerization and organization**		CAPG	0,04	Only in CR
		TPM1	0,04	Only in CR
		TPM3	0,0011	Only in CR
		ZNF185	0,0094	100
		TAGLN2	0,00058	86
		TPM3	0,016	36
		COTL1	0,0022	30
		SEPT2	0,0098	32
		FLNA	0,00017	17
		LASP1	0,00045	14
		TMSB4X	0,021	10
		PFN1	0,00016	6
		ARPC4-TTLL3	0,0084	5,7
		CTTN	0,03	5,5
		MSN	0,00018	4,2
		EZR	0,018	2,8
		ACTN1	0,012	2,6
		CAPZA1	0,0039	4,4
		SPTBN1	0,02	2,2
		SPTAN1	0,037	1,7
		CORO1C	0,0013	0,1
		EPB41L1	0,044	Only in CS
		INF2	0,0045	Only in CS
**Myosin**		MYH10	0,0095	Only in CR
		MYO1C	0,03	69
		MYH9	0,01	9,6
		MYL12A	0,034	8,8
		MYL6	0,011	6
**Tubulins subunits**		TUBB6	0,00036	16
		TUBB	0,0024	3
		TUBA1C	0,0022	2,7
		TUBB4B	0,013	2,4
**Regulation of the MT cytoskeleton polymerization and organization**		STMN1	0,042	Only in CR
		TTLL12	0,014	Only in CR
		SEPT2	0,0098	32
		MAPRE1	0,017	8,6
		TPT1	0,034	2,2
		CORO1C	0,0013	0,1
**Collagen biosynthesis**		PLOD1	0,02	6,6
		COLGALT1	0,005	5,8
**Keratins**	**Type II**	KRT81	0,044	Only in CR
		KRT8	0,039	0,6
		KRT5	0,001	Only in CS
		KRT6A	0,0019	Only in CS
	**Type I**	KRT17	0,029	0,5
		KRT19	0,0026	0,1
		KRT13	0,0064	0,007
		KRT15	< 0,00010	Only in CS
		KRT14	0,00044	Only in CS

	KRT16	0,0012	Only in CS
**Keratin organization**	EPPK1	0,0024	0,2
**E-cadherin/catenin complex**	CTNNB1	0,024	0,1
	CTNNA1	< 0,00010	0,07
	CTNND1	0,00067	0,04
	CDH1	0,0025	Only in CS
**Desmosomal proteins**	JUP	0,037	0,07
	DSG2	0,026	0,06
	DSP	0,00096	0,02
	DSG3	< 0,00010	Only in CS
	PPL	0,026	Only in CS
	PKP3	0,0022	Only in CS
	PKP1	0,0061	Only in CS
**Proteins that form hemidesmosomes**	PLEC	0,0019	0,09
	ITGB4	0,0012	0,02
	ITGA6	0,0011	Only in CS
	LAMA3	< 0,00010	Only in CS
	LAMB3	0,0048	Only in CS
**Other proteins involved in adhesion**	PPFIA1	0,0001	Only in CR
	VCL	0,033	92
	TJP2	0,0025	3,5
	VMP1	0,045	0,3
	SDC1	0,0075	0,1
	BCAM	0,0048	Only in CS
	THBS1	0,017	Only in CS

Table S2. Changes in the level of proteins involved in formation and regulation of cytoskeleton in resistant A-431 cells.

		Gen	T-Test	AR:AS
**Regulation of actin polymerization and organization**		ARPC3	0,036	Only in AR
		SEPT9	0,041	5,4
		LASP1	0,0087	4,1
		TPM1	0,041	3,9
		LCP1	0,035	2,1
		ARPC4-TTLL3	0,034	0,3
		TES	0,00048	Only in AS
		DSTN	0,047	Only in AS
**Tubulin**		TUBA4A	< 0,00010	Only in AR
**Tight junction (TJ)**		CGN	0,00049	Only in AR
**Keratins**	**Type I**	KRT18	0,045	0,6
		KRT13	0,044	0,2
	**Type II**	KRT8	0,018	0,5
		KRT1	0,0071	0,3
		KRT5	0,0049	0,1

Changes in the level of proteins associated with metabolic processes

Table S3. Changes in the level of proteins associated with metabolic processes in resistant CAL-39 cells.

	Gen	T-Test	CR:CS
**Glucose transporter**	SLC2A1	0,013	0,08
**Glycolysis and pyruvate metabolism**	PGAM1	< 0,00010	16
	GLO1	0,0041	11
	PGK1	0,0018	5
	GPI	0,035	5

		TPI1	0,0054	4,8
		LDHA	0,0016	4,4
		ENO1	0,002	2,5
		LDHB	0,0025	2,3
**Tricarboxylic acid) cycle (TCA) cycle**		IDH2	0,012	15
		MDH2	0,00086	0,4
		CS	0,045	0,4
		OGDH	0,029	0,1
**Oxidative phosphorylation**	**Complex V**	ATP5H	0,023	0,5
		ATP5F1A	0,0083	0,4
		ATP5O	0,0075	0,2
	**Complex IV**	COX7A2L	0,049	0,4
		COX5A	0,043	0,2
	**Complex III**	UQCRC2	0,017	0,1
		UQCRFS1	< 0,00010	Only in CS
	**Complex I**	NDUFS1	0,026	0,04
		NDUFA13	0,0028	0,01
		NDUFS4	0,0051	Only in CS
**Nucleotide synthesis nukleotydów**	**Folic acid cycle**	MTHFD1	0,0021	Only in CR
		MTHFD2	0,00039	Only in CS
	**Purine synthesis**	NUDT5	0,015	Only in CR
		IMPDH2	0,002	Only in CR
		GMPS	0,038	100
		ATIC	0,0088	22
	**Synthesis of purines and pyrimidines**	NME1	0,023	5,1
		NME1-NME2	0,026	2
**Heme metabolism**		CPOX	0,012	14
**Glycogenolysis**		PYGL	0,00045	Only in CR
**Pentose Phosphate Pathway**		G6PD	0,00059	0,2
		PGD	0,012	0,05
**S-Adenosyl methionine (SAM) metabolism**		MTAP	0,041	Only in CR
		AHCY	0,0076	17
		PCMT1	0,0031	9,5
**Amino acid synthesis**		PSAT1	0,017	Only in CR
		IDH2	0,012	15
		ASNS	0,0021	12
		MDH1	0,021	8,9
		OAT	0,021	4,4
		PHGDH	0,017	4,3
		GOT2	0,028	0,5
**ß-oxidation of fatty acids**		ACADVL	0,015	2,8
		ACAD9	0,021	0,3
		ACSL3	0,018	0,1
		HSD17B4	0,017	0,3
		SCP2	0,0027	0,2
**Steroid metabolism**		CYB5R3	< 0,00010	4,4
		HINT2	0,00094	0,2
		AKR1D1	0,0022	Only in CS
		AKR1C2	0,022	Only in CS
**Mitochondrial transporters**	**Pi/H+**	SLC25A5	0,029	0,6
	**ADP/ATP**	SLC25A3	0,011	0,3

Table S4. Changes in the level of proteins associated with metabolic processes in resistant A-431 cells.

	Gen	T-Test	AR:AS
**Regulation of acetyl-CoA biosynthesis**	PDP1	< 0,00010	Only in AR
**TCA cycle**	SUCLG2	0,014	Only in AR
	DLD	0,027	1,6
	IDH3B	0,043	0,3
**Respiratory chain (ATP synthases)**	ATP5J	0,042	7,8
	ATP5O	0,027	1,9
**Nucleotide synthesis**	ATIC	0,021	5,2
	AK4	0,0014	0,3
**Lipid metabolism, PpIX and heme transport**	TSPO	0,047	0,3
**Metabolism of ketone bodies**	OXCT1	0,04	0,3

Changes in the level of proteins involved in cellular signaling

Table S5. Changes in the level of proteins involved in cellular signaling in resistant CAL-39 cells.

	Gen	T-Test	CR:CS
**Factors involved in transcription regulation**	STAT3	0,0019	Only in CR
	NUCKS1	0,017	Only in CR
	HDGF	0,0013	15
	RELA	0,03	5,2
	NCOA5	0,036	Only in CS
**Receptors and membrane proteins**	EGFR	0,0031	Only in CR
	LANCL2	0,031	Only in CR
	CD99	0,04	Only in CR
	HLA-B	0,0042	Only in CR
	HLA-A	0,033	15
	GPRC5A	0,034	4
	CD44	0,046	2,5
	CKAP4	0,042	0,4
	TACSTD2	0,0042	0,09
**Kinases**	PRKCI	0,0031	Only in CR
	MAP2K2	0,00061	23
**Phosphatases**	PPM1G	0,01	Only in CR
	PPP2CB	< 0,00010	Only in CR
	PPP2R1A	0,0016	6,1
**Galectins**	LGALS3	0,033	16
	LGALS1	0,0043	5,7
**G proteins**	GNAS	0,031	Only in CS
**Caveolae forming proteins**	CAVIN3	0,0002	50
	CAV1	0,00079	0,2
**S100A proteins**	S100A6	0,0018	6,9
	S100A14	0,018	Only in CS
**Other proteins involved in cellular signaling**	CLIC1	0,0051	Only in CR
	FUT8	0,027	Only in CR
	CDV3	0,0087	20
	PEBP1	0,0011	11
	MIF	0,00071	6,1
	MYOF	0,011	2
	YWHAE	0,031	1,7

Table S6. Changes in the level of proteins involved in cellular signaling in resistant A-431 cells.

	Gen	T-Test	AR:AS
**Transcription factors**	PHF3	0,02	Only in AR
**Receptors**	AXL	0,0041	Only in AR
	EGFR	0,023	0,5
	PTPRF	0,022	0,09
**Kinases**	MAP4K4	0,036	Only in AS
	CSNK2A3	0,024	Only in AS
**G proteins**	GNAI3	0,033	Only in AS
	GNA11	0,037	Only in AS
**Ca^{2+} binding protein**	CALB2	0,00083	Only in AS
**NF-κB regulation, UPR**	DDRGK1	0,041	Only in AS
**Proliferation?**	CDV3	0,015	5

Changes in the level of proteins involved in chromatin regulation

Table S7. Changes in the level of proteins involved in chromatin regulation in resistant CAL-39 cells.

		Gen	T-Test	CR:CS
**Chromatin organization**	**Histones**	HIST1H1C	0,0068	0,5
		HIST1H1E	0,0068	0,5
		HIST1H1D	0,00023	Only in CS
	**Histone binding and/or modification**	TTLL12	0,014	Tylko w CR
		PTMA	0,0092	Tylko w CR
		DEK	0,041	5,2
		CHTOP	0,00023	0,08
		FBLL1	0,02	0,3
		FBL	0,0042	0,4
	**Chromatin-associated non-histone proteins**	HMGB2	0,0059	14
		HMGB1	0,023	5,1
	**Nuclear import of histones and chromatin assembly**	NASP	0,00031	13
		NAP1L1	0,029	2
	**Decompaction of chromatin in response to DNA damage**	PARP1	0,012	4,5
	**Component of DNA helicase**	MCM2	0,042	3,5
	**Nuclear assembly**	TMPO	0,0015	0,4
		BANF1	0,013	0,3

Table S8. Changes in the level of proteins involved in chromatin regulation in resistant A-431 cells.

		Gen	T-Test	AR:AS
**Chromatin organization**	**Histones**	HIST1H1D	0,003	Only in AR
	**DNA helicase**	RUVBL2	0,038	4,1
	**Histone modification**	RUVBL2	0,038	4,1
		PA2G4	0,039	4
		TRIM28	0,047	0,7
		HAT1	0,02	0,2
	**Chromatin silencing**	UTP3	0,02	0,06

Changes in the level of proteins involved in cell cycle

Table S9. Changes in the level of proteins involved in cell cycle in resistant CAL-39 cells.

		Gen	T-Test	CR:CS
**Cell cycle**	**Replication**	NASP	0,00031	13
		MCM2	0,042	3,5
		NAP1L1	0,029	2
	**Centrosome duplication**	CHORDC1	0,013	Only in CR
	**Centrosome cohesion before mitosis**	CROCC	0,033	5
	**Mitosis**	NUDC	0,00042	Only in CR
		SEPT2	0,0098	32
		CLTA	0,008	9
		NUMA1	0,0026	0,04
	**Cytokinesis**	NUDC	0,00042	Only in CR
		PDCD6IP	0,014	Only in CR
		SEPT2	0,0098	32
		CHMP4B	0,014	8,8

Table S10. Changes in the level of proteins involved in cell cycle in resistant A-431 cells.

		Gen	T-Test	AR:AS
**Cell cycle**	**Replication**	MCM3	0,033	2
	**Centrosome cohesion before mitosis**	CROCC	0,016	2
	**Cytokinesis**	SEPT9	0,041	5,4

Changes in the level of proteins that may be involved in immunogenic cell death

Table S11. Changes in the level of proteins that may be involved in immunogenic cell death in resistant CAL-39 cells.

		Gen	T-Test	CR:CS
**MHC class I**		HLA-B	0,0042	Only in CR
		HLA-A	0,033	15
**Escape from immune response**		LGALS3	0,033	16
		LGALS1	0,0043	5,7
		MIF	0,00071	6,1
**Proteins that may act as DAMPS**	**HSP70**	HSPA2	0,017	5
		HMGB1	0,012	4,5
	**HSP90**	HSP90AB2P	0,025	2,7
		HSP90AB1	0,011	2,2
		HSP90AA1	0,032	1,9
		CALR	0,016	1,9
**TF involved in immune response**		STAT3	0,0019	Only in CR
		RELA	0,03	5,2
**Antigen processing**		ERAP1	0,0058	0,1
		HM13	0,0083	0,02

Table S12. Changes in the level of proteins involved in antigen processing in resistant A-431 cells.

	Gen	T-Test	AR:AS
**Antigen processing**	TAP1	0,032	0,3

	TAPBP	0,0011	0,08
	HM13	0,035	0,2

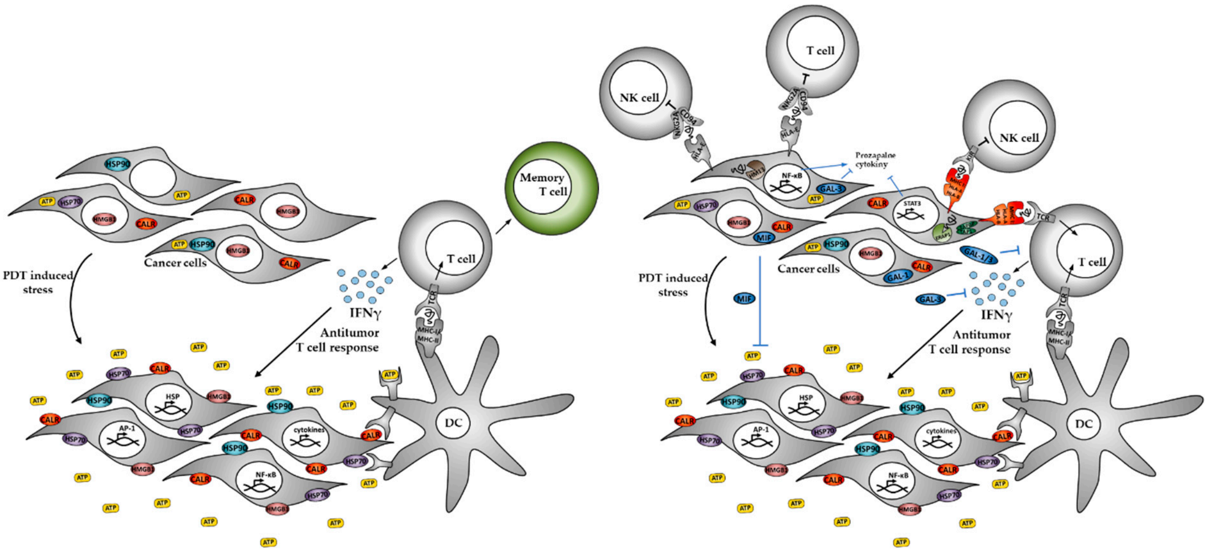

Figure S1 Immunogenic cell death and resistance to PDT. (**a**) Immunogenic cell death induced by certain anti-cancer therapies, including PDT, due to stress and exposure/release of DAMP (e.g., CALR, ATP, HMGB1, HSP90, HSP70, tumor-derived nucleic acids), where the immune system is activated and triggers immunity against dead-cell antigens [18-22]. (**b**) Effect of detected changes in PDT-resistant cells on the immune system. An increased number of proteins that can act as DAMP and molecules of MHC class I. An increased level of NF-κB, which may promote the initiation of an anti-tumor response while decreased level of proteins involved in the processing of antigens for MHC-I, increased amount of STAT3, MIF and galectins which may lead to the escape of cancer cells from immune system. Presentation of antigens by HLA-E is also important for the antitumor response.

Changes in the level of proteins involved in antioxidant defense

Table S13. Changes in the level of proteins involved in antioxidant defense in resistant CAL-39 cells.

		Gen	T-Test	CR:CS
**Antioxidant defense**	**Glutathione S-transferases**	GSTO1	< 0,00010	Only in CR
		MGST2	< 0,00010	Only in CS
	**Antioxidant and detoxification activity**	TST	0,035	Only in CR
		FAM213A	0,045	0,3
	**Protection against oxidative stress-induced cell-death**	SLC25A24	0,0051	3,3
		TPT1	0,034	2,2
	**Degradation of oxidatively damaged polypeptides**	LONP1	0,026	2,9
	**Thiol-specific peroxidases**	PRDX3	0,011	3
		PRDX4	0,0071	2,7
		PRDX6	0,013	2,4
	**Thioredoxin**	TXN	0,00095	0,3

Changes in the level of proteins involved in cellular transport

Table S14. Changes in the level of proteins involved in vesicular transport in resistant CAL-39 cells.

			Gen	T-Test	CR:CS
Vesicular transport	RAB activity regulation		GDI1	< 0,00010	Only in CR
			GDI2	0,0048	30
	Endocytosis	Clathrin-dependent	SCAMP1	0,00014	Only in CR
			CLTA	0,008	9
		Caveolae	CAVIN3	0,0002	50
			CAV1	0,00079	0,2
			RAB5C	0,01	4,9
		Recycling of transmembrane proteins	TXLNA	0,024	Only in CR
			VPS26A	0,021	Only in CR
			VPS35	0,035	13
		multivesicular bodies (MVB)	PDCD6IP	0,014	Only in CR
			CHMP4B	0,014	8,8
	Exocytosis		MAGEA4	< 0,00010	32
			MVP	0,039	16
			CHP1	0,0046	0,09
	From ER to Golgi complex		SEC24C	0,00097	Only in CR
			SEC31A	0,0059	4
			TMED5	0,029	0,2
	MPR transport		PLIN3	0,0018	45
	Intracellular nanovesicles (INV)		TPD52L2	0,026	2,5
	Microtubule based movement		KTN1	0,0046	0,5
	From Golgi apparatus to ER		RAB6A	0,027	0,3
Nuclear transport			IPO5	0,033	Only in CR
			NUP205	0,035	Only in CR
			XPO1	0,037	7
			RCC1	0,0084	0,05

Changes in the level of proteins involved in RNA processing

Table S15. Changes in the level of proteins involved in RNA processing in resistant CAL-39 cells.

	Gen	T-Test	CR:CS
RNA processing	SSB	< 0,00010	Only in CR
	EDC4	0,0056	Only in CR
	CCDC12	0,019	Only in CR
	NSUN2	0,033	Only in CR
	DNAJC8	0,038	Only in CR
	HSPA2	0,017	5
	EFTUD2	0,0046	3,6
	DDX17	0,032	1,5
	SYNCRIP	0,013	0,5
	HNRNPD	0,036	0,5
	HNRNPA1	0,0019	0,4
	THOC6	0,032	0,4
	FBL	0,0042	0,4
	FBLL1	0,02	0,3
	SNRPB	0,046	0,1
	HNRNPUL1	0,014	0,1
	CHTOP	0,00023	0,08

	HNRNPDL	0,0051	0,04
	SRSF9	0,021	Only in CS

Table S16. Changes in the level of proteins involved in RNA processing in resistant A-431 cells.

	Gen	T-Test	AR:AS
**RNA processing**	PPAN-P2RY11	0,0036	Only in AR
	SF3A2	0,05	Only in AR
	MBNL1	0,019	5,9
	PA2G4	0,039	4
	SNU13	0,035	2,4
	MAGOHB	0,021	2,1
	SNRPE	0,023	0,6
	NONO	0,035	0,5
	PCBP1	0,028	0,4
	SNRPC	0,018	0,2
	DDX1	0,05	0,2
	HNRNPK	0,0026	Only in AS

Mitochondrial respiration comparison in A-431 resistant and sensitive cell

Changes in the level of proteins involved in formation and regulation of cytoskeleton

As changes in the level of proteins involved in metabolism in A-431 cells were not as clear as in CAL-39 cell lines, comparison of mitochondrial respiration was performed on whole sensitive and resistant cells of A-431 cells with the use of Oroboros. Resistant A-431 cells showed an increase in basal respiration, O_2 consumption for ATP production, and maximum respiration capacity.

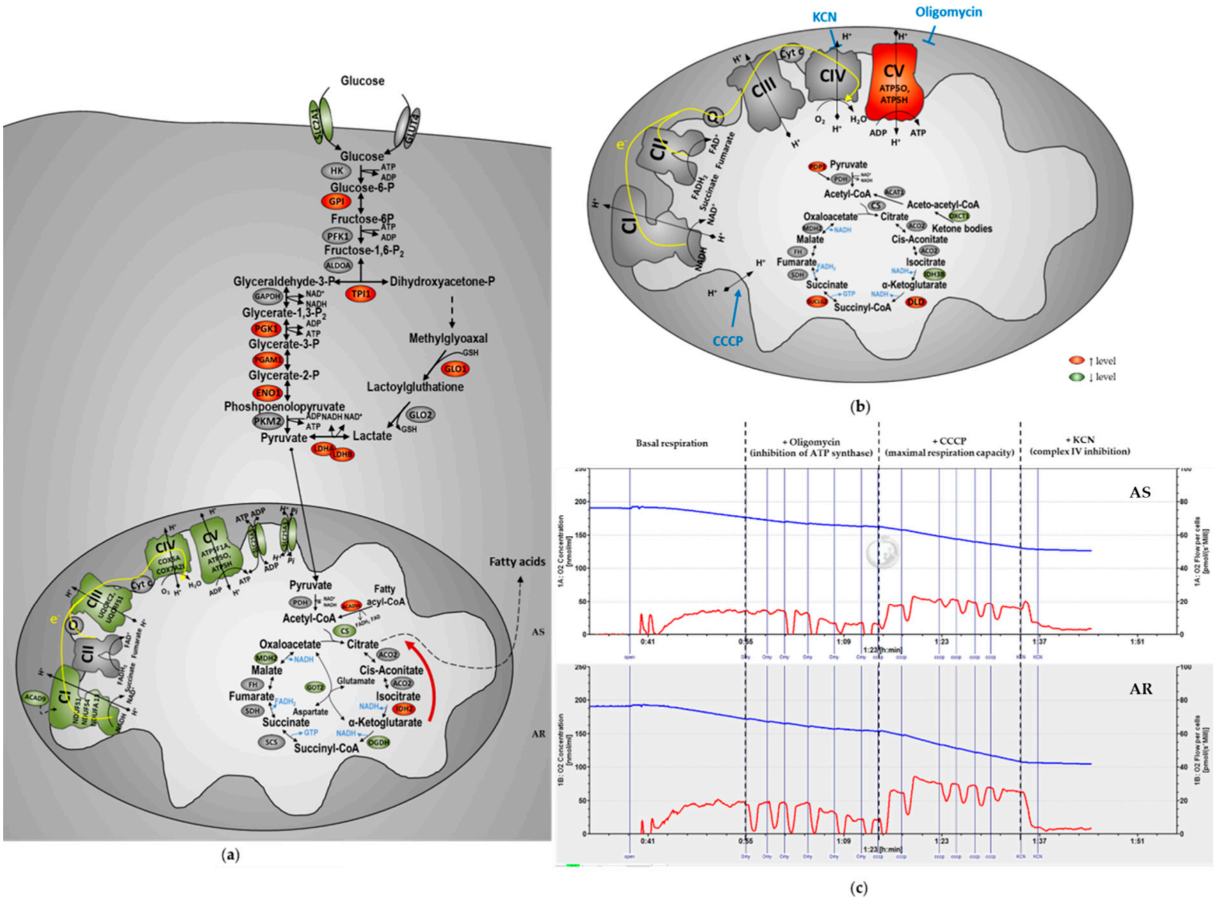

Figure S2. Mitochondrial respiration (**a**) Warburg effect in resistant CAL-39 cells. Despite the reduced amount of

the constitutive glucose transporter 1 (SLC2A1), these cells elevated level of proteins that may lead to increased glucose uptake (LANCL2 [16], NUCKS1 [23,24], PRKCι [25], 14-3-3 [26]), e.g., as a result of activation of signaling pathways leading to GLUT4 transport to the cell membrane. These cells also had increased level of glycolytic pathway enzymes and decreased level of oxidative phosphorylation enzymes; (**b**) changes in resistant A-431 cells and mechanism of action of CCCP, oligomycin and KCN. Resistant A-431 cells were characterized by a higher level of mitochondria and enzymes involved in aerobic respiration; (**c**) comparison of mitochondrial respiration in sensitive and resistant A-431 cells. Resistant cells characterize a higher basal respiration, O_2 consumption to ATP production and maximal respiratory capacity.

References:

1. Ruggieri, V.; Agriesti, F.; Tataranni, T.; Perris, R.; Mangieri, D. Paving the Path for Invasion: The Polyedric Role of LASP1 in Cancer. *Tumour Biol.* **2017**, *39*, 1–6, doi:10.1177/1010428317705757.
2. Butt, E.; Raman, D. New Frontiers for the Cytoskeletal Protein LASP1. *Frontiers in Oncology* **2018**, *8*, 1–11.
3. Zhang, X.; Liu, Y.; Fan, C.; Wang, L.; Li, A.; Zhou, H.; Cai, L.; Miao, Y.; Li, Q.; Qiu, X.; et al. Lasp1 Promotes Malignant Phenotype of Non-Small-Cell Lung Cancer via Inducing Phosphorylation of FAK-AKT Pathway. *Oncotarget* **2017**, *8*, 75102–75113, doi:10.18632/oncotarget.20527.
4. Li, M.; Jin, C.; Xu, M.; Zhou, L.; Li, D.; Yin, Y. Bifunctional Enzyme ATIC Promotes Propagation of Hepatocellular Carcinoma by Regulating AMPK-MTOR-S6 K1 Signaling. *Cell Commun Signal* **2017**, *15*, 52–52, doi:10.1186/s12964-017-0208-8.
5. Shi, Z.; Zhang, J.; Zheng, S. What We Know about ST13, a Co-Factor of Heat Shock Protein, or a Tumor Suppressor? *J Zhejiang Univ Sci B* **2007**, *8*, 170–176, doi:10.1631/jzus.2007.B0170.
6. Májek, P.; Reicheltová, Z.; Štikarová, J.; Suttnar, J.; Sobotková, A.; Dyr, J.E. Proteome Changes in Platelets Activated by Arachidonic Acid, Collagen, and Thrombin. *Proteome Sci* **2010**, *8*, 1–13, doi:10.1186/1477-5956-8-56.
7. Xiao, H.; Zhou, B.; Jiang, N.; Cai, Y.; Liu, X.; Shi, Z.; Li, M.; Du, C. The Potential Value of CDV3 in the Prognosis Evaluation in Hepatocellular Carcinoma. *Genes & Diseases* **2018**, *5*, 167–171, doi:10.1016/j.gendis.2018.01.003.
8. Kochan, G.; Escors, D.; Breckpot, K.; Guerrero-Setas, D. Role of Non-Classical MHC Class I Molecules in Cancer Immunosuppression. *OncoImmunology* **2013**, *2*, e26491-1–8, doi:10.4161/onci.26491.
9. Zheng, H.-C. The Molecular Mechanisms of Chemoresistance in Cancers. *Oncotarget* **2017**, *8*, 59950–59964, doi:10.18632/oncotarget.19048.
10. Chen, C.; Zhao, S.; Karnad, A.; Freeman, J.W. The Biology and Role of CD44 in Cancer Progression: Therapeutic Implications. *Journal of Hematology & Oncology* **2018**, *11*, 1–23, doi:10.1186/s13045-018-0605-5.
11. Toole, B.P. Hyaluronan-CD44 Interactions in Cancer: Paradoxes and Possibilities. *Clin Cancer Res* **2009**, *15*, 7462–7468, doi:10.1158/1078-0432.CCR-09-0479.
12. Xu, H.; Tian, Y.; Yuan, X.; Wu, H.; Liu, Q.; Pestell, R.; Wu, K. The Role of CD44 in Epithelial–Mesenchymal Transition and Cancer Development. *OTT* **2015**, *8*, 3783–3792, doi:10.2147/OTT.S95470.
13. Manara, M., Cristina; Pasello, M.; Scotlandi, K. CD99: A Cell Surface Protein with an Oncojanus Role in Tumors. *Genes* **2018**, *9*, 1–17, doi:10.3390/genes9030159.
14. Zhou, H.; Rigoutsos, I. The Emerging Roles of GPRC5A in Diseases. *Oncoscience* **2014**, *1*, 765–776, doi:10.18632/oncoscience.104.
15. Jiang, X.; Xu, X.; Wu, M.; Guan, Z.; Su, X.; Chen, S.; Wang, H.; Teng, L. GPRC5A: An Emerging Biomarker in Human Cancer. *BioMed Research International* **2018**, *2018*, 1–11, doi:10.1155/2018/1823726.
16. Zocchi, E.; Hontecillas, R.; Leber, A.; Einerhand, A.; Carbo, A.; Bruzzone, S.; Tubau-Juni, N.; Philipson, N.; Zoccoli-Rodriguez, V.; Sturla, L.; et al. Abscisic Acid: A Novel Nutraceutical for Glycemic Control. *Front. Nutr.* **2017**, *4*, 1–13, doi:10.3389/fnut.2017.00024.
17. Zhu, C.; Wei, Y.; Wei, X. AXL Receptor Tyrosine Kinase as a Promising Anti-Cancer Approach: Functions, Molecular Mechanisms and Clinical Applications. *Mol Cancer* **2019**, *18*, 1–22, doi:10.1186/s12943-019-1090-3.
18. Agostinis, P.; Berg, K.; Cengel, K.A.; Foster, T.H.; Girotti, A.W.; Gollnick, S.O.; Hahn, S.M.; Hamblin, M.R.; Juzeniene, A.; Kessel, D.; et al. Photodynamic Therapy of Cancer: An Update. *CA: A Cancer Journal for Clinicians* **2011**, *61*, 250–281, doi:10.3322/caac.20114.
19. Galluzzi, L.; Vitale, I.; Aaronson, S.A.; Abrams, J.M.; Adam, D.; Agostinis, P.; Alnemri, E.S.; Altucci, L.; Amelio, I.; Andrews, D.W.; et al. Molecular Mechanisms of Cell Death: Recommendations of the

Nomenclature Committee on Cell Death 2018. *Cell Death Differ* **2018**, *25*, 486–541, doi:10.1038/s41418-017-0012-4.

20. Krysko, D.V.; Garg, A.D.; Kaczmarek, A.; Krysko, O.; Agostinis, P.; Vandenabeele, P. Immunogenic Cell Death and DAMPs in Cancer Therapy. *Nat Rev Cancer* **2012**, *12*, 860–875, doi:10.1038/nrc3380.
21. Tang, D.; Kang, R.; Berghe, T.V.; Vandenabeele, P.; Kroemer, G. The Molecular Machinery of Regulated Cell Death. *Cell Res* **2019**, *29*, 347–364, doi:10.1038/s41422-019-0164-5.
22. van Straten, D.; Mashayekhi, V.; de Bruijn, H.; Oliveira, S.; Robinson, D. Oncologic Photodynamic Therapy: Basic Principles, Current Clinical Status and Future Directions. *Cancers* **2017**, *9*, 1–54, doi:10.3390/cancers9020019.
23. Huang, P.; Cai, Y.; Zhao, B.; Cui, L. Roles of NUCKS1 in Diseases: Susceptibility, Potential Biomarker, and Regulatory Mechanisms. *BioMed Research International* **2018**, *2018*, 1–7, doi:10.1155/2018/7969068.
24. Chang, L.; Chiang, S.-H.; Saltiel, A.R. Insulin Signaling and the Regulation of Glucose Transport. *Mol Med* **2004**, *10*, 65–71, doi:10.2119/2005-00029.Saltiel.
25. Nomiyama, R.; Emoto, M.; Fukuda, N.; Matsui, K.; Kondo, M.; Sakane, A.; Sasaki, T.; Tanizawa, Y. Protein Kinase C Iota Facilitates Insulin-induced Glucose Transport by Phosphorylation of Soluble NSF Attachment Protein Receptor Regulator (SNARE) Double C2 Domain Protein b. *J Diabetes Investig* **2019**, *10*, 591–601, doi:10.1111/jdi.12965.
26. Pennington, K.; Chan, T.; Torres, M.; Andersen, J. The Dynamic and Stress-Adaptive Signaling Hub of 14-3-3: Emerging Mechanisms of Regulation and Context-Dependent Protein–Protein Interactions. *Oncogene* **2018**, *37*, 5587–5604, doi:10.1038/s41388-018-0348-3.
27. Mossakowska, B.J.; Shahmoradi Ghahe, S.; Cysewski, D.; Fabisiewicz, A.; Tudek, B.; Siedlecki, J.A. Mechanisms of Resistance to Photodynamic Therapy (PDT) in Vulvar Cancer. *IJMS* **2022**, *23*, 4117, doi:10.3390/ijms23084117.
